# Small molecule regulated dynamic structural changes of human G-quadruplexes†
†Electronic supplementary information (ESI) available: Experimental details, synthetic procedures, characterization data of compounds, ^1^H NMR and ^13^C NMR spectra, sm-FRET, shot noise, FCS-FRET data, lifetime data, CD spectra, NMR spectra. See DOI: 10.1039/c6sc00057f


**DOI:** 10.1039/c6sc00057f

**Published:** 2016-02-12

**Authors:** Manish Debnath, Shirsendu Ghosh, Deepanjan Panda, Irene Bessi, Harald Schwalbe, Kankan Bhattacharyya, Jyotirmayee Dash

**Affiliations:** a Department of Organic Chemistry , Indian Association for the Cultivation of Science , Jadavpur , Kolkata-700032 , India . Email: ocjd@iacs.res.in; b Department of Physical Chemistry , Indian Association for the Cultivation of Science , Jadavpur , Kolkata-700032 , India; c Institute of Organic Chemistry and Chemical Biology , Goethe University Frankfurt and Center for Biomolecular Magnetic Resonance (BMRZ) , Max-von-Laue Strasse 7 , 60438 , Frankfurt am Main , Germany

## Abstract

The changes in structure and dynamics of oncogenic (*c-MYC*) and telomeric (*h-TELO*) G-rich DNA sequences due to the binding of a novel carbazole derivative (**BTC**) are elucidated using single-molecule Förster resonance energy transfer (sm-FRET), fluorescence correlation spectroscopy (FCS) and NMR spectroscopy. In contrast to the previous reports on the binding of ligands to pre-folded G-quadruplexes, this work illustrates how ligand binding changes the conformational equilibria of both unstructured G-rich DNA sequences and K^+^-folded G-quadruplexes. The results demonstrate that K^+^ free *c-MYC* and *h-TELO* exist as unfolded and partially folded conformations. The binding of **BTC** shifts the equilibria of both investigated DNA sequences towards the folded G-quadruplex structure, increases the diffusion coefficients and induces faster end-to-end contact formation. **BTC** recognizes a minor conformation of the *c-MYC* quadruplex and the two-tetrad basket conformations of the *h-TELO* quadruplex.

## Abstract 2

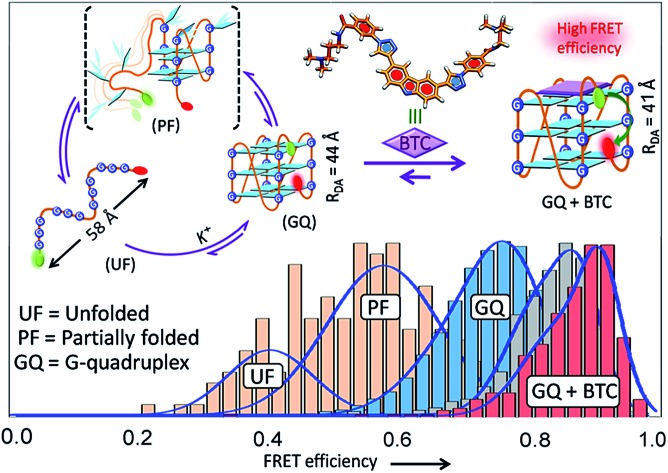
A carbazole derivative (**BTC**) regulates the dynamics of unstructured human *c-MYC* and *h-TELO* sequences by folding them into compact quadruplex structures.

## Introduction

Ligand-mediated stabilization of G-quadruplexes is considered a promising strategy for the development of anti-cancer therapeutics as well as DNA-nanotechnology.^1^–^6^ However, little is known about how ligands can affect the conformational dynamics of G-quadruplexes. G-quadruplexes are associated with the transcriptional regulation of various oncogenes (*c-MYC*,^7^*c-KIT1*,^8^*c-KIT2* (ref. 9) *etc*.) and telomere maintenance.^10^ Consequently, small molecules that can bind G-quadruplexes are considered potential anticancer agents.^1^–^5^,^10^ Small molecule-induced stabilization of G-quadruplexes with or without cations has been studied by a variety of biophysical techniques.^11^–^18^ Most of the previous studies report the interaction of pre-folded static G-quadruplexes with small molecules. Human G-quadruplexes are, however, highly dynamic.^19^ Characterizing the conformational equilibria of unstructured G-rich DNA sequences in the presence and absence of small molecules might prove useful for innovative drug design.

Single-molecule Förster resonance energy transfer (sm-FRET) is a powerful method that provides key information about the structure, population distribution of folded or unfolded species and the end-to-end distance of bio-molecules.^19^–^35^ It has been used to investigate the effect of binding of either protein^26^ or metal ions (K^+^/Na^+^)^27^–^29^ on the conformational dynamics of G-quadruplexes. However, only a few studies have been conducted to elucidate the influence of small molecules on the conformational dynamics of G-quadruplex DNA.^30^,^31^

Fluorescence correlation spectroscopy (FCS) tracks single or several molecules in solution to investigate changes in diffusion coefficients, molecular size and intra-molecular contact dynamics.^32^–^38^ FCS has been used to investigate the intra-molecular dynamics of a DNA hairpin tagged with a donor–acceptor FRET pair.^36^–^38^ A combination of sm-FRET and FCS has been used by Majima and co-workers to quantitatively analyse the pH-induced intra-molecular folding dynamics of an i-motif DNA.^39^ We herein illustrate the changes in structure and dynamics in unstructured and K^+^-folded *c-MYC* and *h-TELO* DNA G-quadruplex forming sequences triggered by a G-quadruplex binding ligand bis-triazolylcarbazole^40^ (**BTC**) using a combination of sm-FRET and FCS. Interaction of **BTC** with G-quadruplexes has also been substantiated by FRET melting, circular dichroism, fluorescence lifetime and NMR spectroscopy studies.

## Results and discussion

### BTC induced stabilization of G-quadruplex forming sequences in the absence of K^+^

**Fig. 1:**
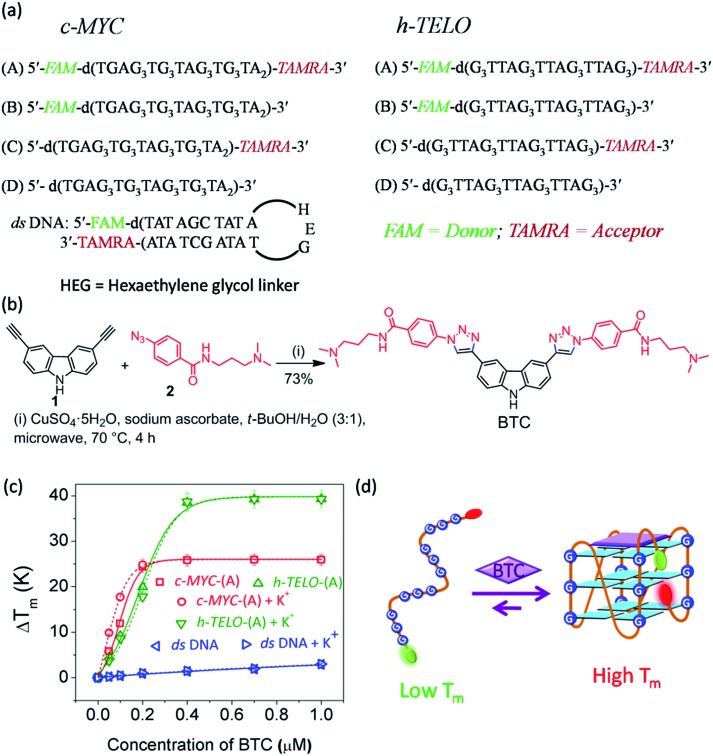
(a) DNA sequences (*c-MYC*, *h-TELO* and *ds* DNA) used in this study, (b) one-pot synthesis of carbazole derivative **BTC**, (c) FRET melting profiles of *c-MYC*-(A), *h-TELO*-(A) and *ds* DNA with increasing [**BTC**] in the presence and absence of K^+^; *T*_m_ (°C) for *c-MYC*-(A) = (69 ± 1), *h-TELO*-(A) = (55 ± 1), and *ds* DNA = (57 ± 1). (d) Schematic representations of a G-quadruplex formation by **BTC** induced folding of a G-rich strand.

FRET melting analysis determines ligand-induced stabilization of folded G-quadruplex structures.^41^ We have examined the effect of **BTC** on the melting profile of both folded and unfolded *c-MYC* and *h-TELO* G-quadruplexes. We have used dual labeled *c-MYC*-(A) and *h-TELO*-(A) quadruplexes and a hairpin duplex (*ds*) DNA control with FAM–TAMRA donor (D)–acceptor (A) FRET pairs (Fig. 1a).

Following our recently developed procedure, **BTC** was prepared using a one-pot Cu(i) catalyzed azide and alkyne cycloaddition in high yields^40^ (Fig. 1b, and S1, ESI†). FRET melting revealed that **BTC** exhibits maximum stabilization potentials for K^+^-folded *c-MYC*-(A) and *h-TELO*-(A) (*i*.*e*. pre-annealed in K^+^ buffer) with Δ*T*_m_ values of 24.0 °C and 38.7 °C, respectively. It is important to note that **BTC** shows a similar increase in the *T*_m_ values of unfolded *c-MYC*-(A) and *h-TELO*-(A) sequences (Fig. 1c and Table S1, ESI†). These results indicate that **BTC** can stabilize *c-MYC*-(A) and *h-TELO*-(A) G-quadruplexes even in the absence of K^+^ ions (Fig. 1d).^40^ Furthermore, **BTC** is found to be selective for G-quadruplexes over duplex DNA as it did not significantly alter the *T*_m_ value of duplex (*ds*) DNA (Table S1, ESI†).

### **BTC** induced dynamic transitions between the ensembles of unfolded conformations to the folded state in G-quadruplexes

We then measured the sm-FRET between the donor–acceptor fluorophores to monitor the conformational changes in K^+^-free and K^+^-folded *c-MYC*-(A) and *h-TELO*-(A) sequences induced by the binding of **BTC**.

**Fig. 2:**
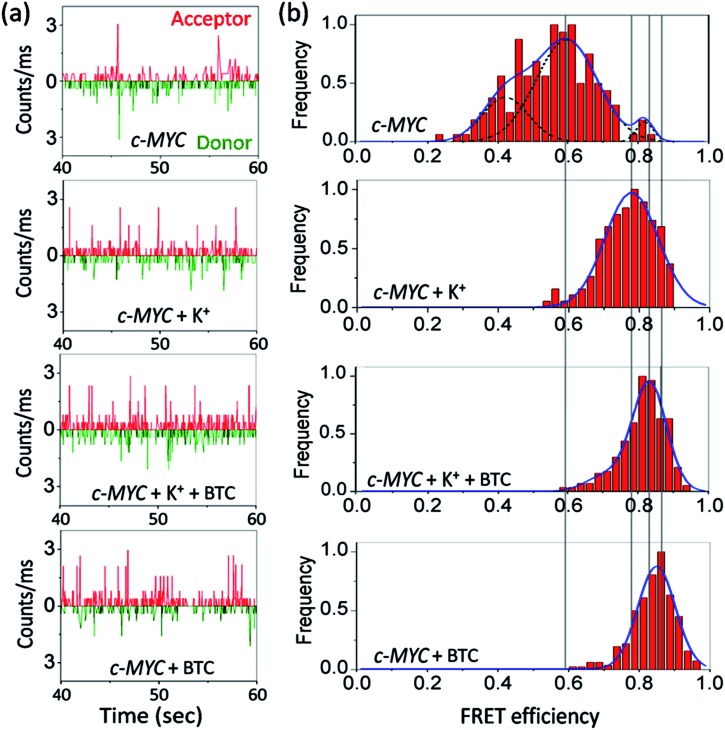
Photon bursts of donor/acceptor (background corrected) (a), and FRET efficiency distributions (b) of 100 pM dual fluorescently labeled *c-MYC*-(A) G-quadruplex forming sequence in the presence and absence of K^+^ and **BTC**. For detailed information see Fig. S4 and S5, ESI.†

Dual labeled sequences of the highest purity are used to exclude the signals from the donor only sample and no appreciable donor bleaching is observed under current experimental conditions (Fig. S2 and S3, ESI†). We observed that the donor–acceptor fluorescence intensities of the dual-labeled G-rich sequences produce anti-correlated fluctuations in the presence and absence of **BTC** and K^+^ ions (Fig. 2 and S4, ESI†). The histograms obtained from the time traces were fitted with tri- and single Gaussian distributions (Fig. 2b and S5, ESI†). The contributions of shot noise in each FRET peak are determined (Table S2, ESI†). The analysis of the FRET histogram of K^+^-free *c-MYC*-(A) shows two major peaks with FRET efficiencies (*ε*_FRET_) centered at ∼0.4 (30%) and 0.6 (68%) (Table S3, ESI†). The distances between donor and acceptor (*R*_DA_) dyes corresponding to each FRET state (0.4 and 0.6) calculated using eqn S3 (ESI†), are 58.4 Å and 51.7 Å, respectively. The FRET histogram of the K^+^-folded *c-MYC*-(A) shows only one major peak at ∼0.8 (97%). Upon addition of **BTC** (1 equiv.), the single peak at ∼0.8 is preserved in the histogram (Fig. 2b). The average *R*_DA_ determined for the K^+^-folded *c-MYC*-(A) in the presence and absence of **BTC** lies between ∼44–42 Å. Interestingly, the FRET distribution of the K^+^-free *c-MYC*-(A) in the presence of **BTC** exhibits a narrow peak (Fig. 2b) with higher efficiency (*ε*_FRET_ ∼ 0.85) corresponding to a *R*_DA_ value of ∼41 Å.

The FRET histogram of K^+^-free *h-TELO*-(A) shows a wide distribution (mean *ε*_FRET_ ∼ 0.51) with a *R*_DA_ of ∼54.6 Å (Fig. S4, ESI†). The K^+^-folded *h-TELO*-(A) shows a *ε*_FRET_ value of ∼0.8 with a *R*_DA_ of ∼45 Å. Upon addition of **BTC** to either K^+^-free or the K^+^-folded *h-TELO*-(A), the *ε*_FRET_ distribution is shifted towards a higher value (∼0.9). The corresponding *R*_DA_ values were determined to be ∼40.6 Å (**BTC** and K^+^) and ∼39 Å (only **BTC**). Considering the presence of extra nucleotides and the dye linkers present in the dual labeled sequences, the *R*_DA_ values obtained for *c-MYC*-(A) and *h-TELO*-(A) in the presence of K^+^ and **BTC** (∼39–42 Å), are close to the size of a folded G-quadruplex [diameter ∼ 25 Å] conformation.

In unfolded structures, the distances between the donor and acceptor fluorophores (*R*_DA_ ∼ 54–58 Å) are large and, consequently, the *ε*_FRET_ values are small. The low FRET peak observed for the K^+^-free *c-MYC*-(A) sequence at *ε*_FRET_ ∼0.4 may be assigned to the unfolded (single stranded) structure. The FRET state ∼0.6 indicates the existence of an intermediate state, presumably an ensemble of partially folded structures. The high FRET peak (∼0.8–0.9) indicates the formation of G-quadruplex structures.

**BTC**-induced quadruplex formation is further corroborated by carrying out sm-FRET experiments using a dual labeled mutant *c-MYC* sequence (Fig. S6 and S7, ESI†). Upon addition of **BTC**, the FRET histogram of *c-MYC*-mut doesn't show any significant change in FRET efficiency. These results suggest that G-rich sequences primarily remain as unfolded and partially folded structures in the absence of **BTC** and K^+^. **BTC** apparently induces and shifts the equilibrium to a folded G-quadruplex conformation as evidenced by the shift of FRET histograms towards higher values.

### FCS measured ligand induced conformational fluctuations in G-quadruplex structures

**Fig. 3:**
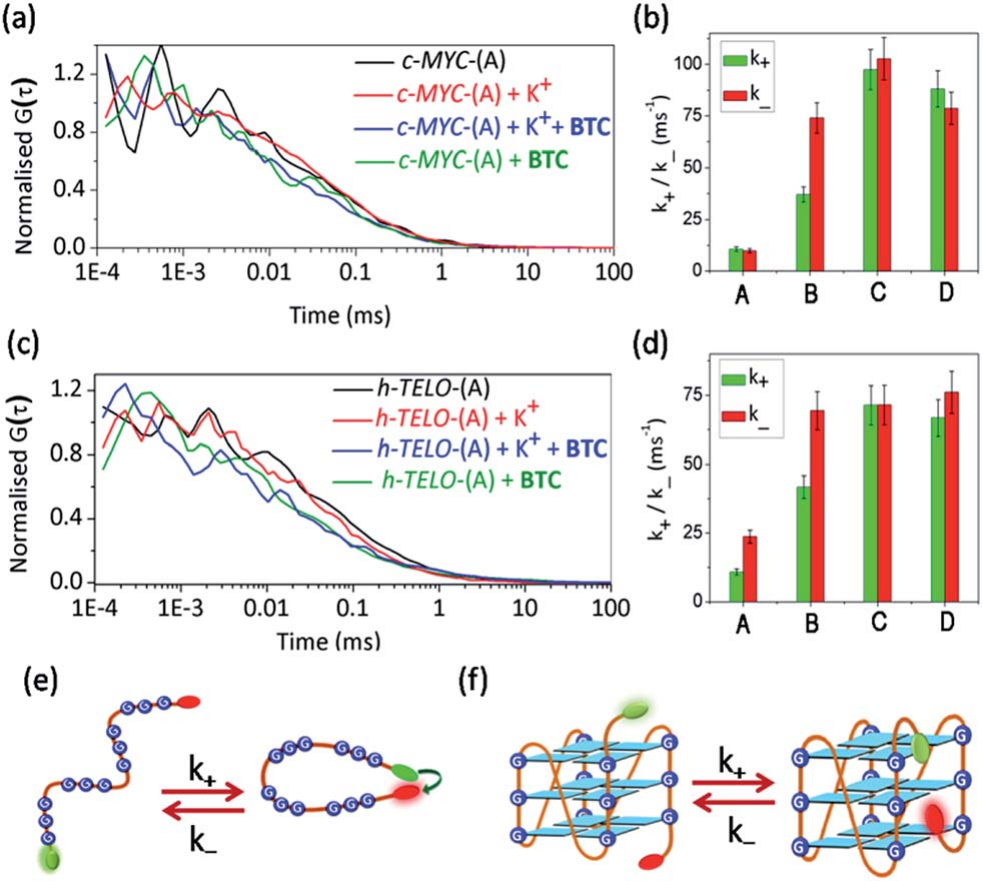
The autocorrelation traces of FAM–TAMRA dual labeled (a) *c-MYC*-(A) in the presence and absence of K^+^ and **BTC**. (b) Plots representing change in rate constant for donor–acceptor contact formation (*k*_+_) and dissociation (*k*_–_) values of K^+^-free *c-MYC*-(A) [lane A]; K^+^-folded *c-MYC*-(A) [lane B]; **BTC** + K^+^-folded *c-MYC*-(A) [lane C]; **BTC** + K^+^-free *c-MYC*-(A) [lane D]. (c) The autocorrelation traces of FAM–TAMRA dual labeled *h-TELO*-(A) in the presence and absence of K^+^ and **BTC**. (d) Plots representing change in *k*_+_ and *k*_–_ values in K^+^-free *h-TELO*-(A) [lane A]; K^+^-folded *h-TELO*-(A) [lane B]; **BTC** + K^+^-folded *h-TELO*-(A) [lane C]; **BTC** + K^+^-free *h-TELO*-(A) [lane D]. Schemes showing the end-to-end contact formation in (e) unfolded DNA and (f) folded DNA.

In addition to sm-FRET, a combination of FCS with FRET is used to investigate the ligand-induced diffusion properties of *c-MYC*-(A) and *h-TELO*-(A) sequences. The fluorescence fluctuations due to the diffusion and inter-conversion of the dual labeled G-rich sequences between the folded (high FRET) and unfolded (low FRET) states are analysed as they pass through the focal volume of the microscope. The resulting FCS curves are shown in Fig. 3a and c (Fig. S8, ESI†). In FCS, the observed fluctuations in fluorescence intensity are expressed as time-correlation functions, *G*(*τ*)^39^ (eqn (1)).1
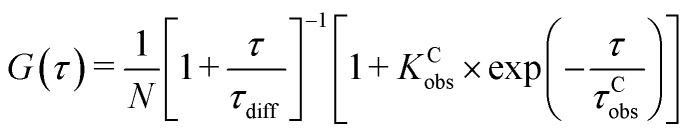
Where *N* is the average number of labeled DNA sequences in the observed volume, *τ*_D_ is the molecular diffusion time, *K*Cobs is the observed amplitude of the donor–acceptor contact formation kinetics due to changes of the donor–acceptor distance, and *τ*Cobs is the observed time of donor–acceptor contact formation.

The diffusion coefficients (*D*_t_) were calculated from the diffusion time (*τ*_D_) using equation S8, ESI† (Tables 1 and S4, ESI†). The *D*_t_ of K^+^-free *c-MYC*-(A) is 221 μm^2^ s^–1^ and increases to 296 μm^2^ s^–1^ in the K^+^-folded conformation. Similarly, the *D*_t_ of the K^+^-free *h-TELO*-(A) increases in the K^+^-folded conformations (Tables 1 and S4, ESI†). Addition of **BTC** to the K^+^-folded *c-MYC*-(A) and *h-TELO*-(A) results in further increase in the *D*_t_ values to 314 μm^2^ s^–1^ and 318 μm^2^ s^–1^, respectively. It is intriguing to observe that the binding of **BTC** to the K^+^-free *c-MYC*-(A) and *h-TELO*-(A) increases the *D*_t_ values to 318 μm^2^ s^–1^ and that is ∼43% and ∼32% higher compared to the K^+^-free *c-MYC*-(A) and *h-TELO*-(A) sequences. According to the Stokes–Einstein eqn (S10, ESI†), the diffusion coefficient is inversely related to the hydrodynamic radii (*R*_h_) for a freely diffusing molecule. Therefore, the observed increase in *D*_t_ of the K^+^-free *c-MYC*-(A) and *h-TELO*-(A) sequences can be attributed to a ∼43% and ∼32% decrease in *R*_h_ upon the binding of **BTC**. The decrease in *R*_h_ estimated theoretically from the ratio of frictional coefficients (*f*/*f*_0_) indicates that the binding of **BTC** to the K^+^-free *c-MYC*-(A) and *h-TELO*-(A) leads to a ∼50% reduction in the *R*_h_ values. In addition, the diffusion parameters calculated using eqn S19, ESI† are quite similar to those obtained from the simplified eqn (1) (Table S5, ESI†). To further validate our findings, we carried out parallel diffusion experiments with single (FAM) labeled *c-MYC*-(B) and *h-TELO*-(B) G-quadruplex forming sequences (Fig. S9, ESI†). The τ_D_ values for single labeled *c-MYC*-(B) and *h-TELO*-(B) in the presence and absence of **BTC** and K^+^ ions are similar to those of the dual labeled *c-MYC*-(A) and *h-TELO*-(A) sequences. Hence the covalent attachment of the dyes does not significantly impact the diffusion parameters (Fig. S9 and Table S6, ESI†).

**Table 1:** Table representing diffusion coefficients (*D*_t_), observed time of intra-chain contact formation (*τ*Cobs), and rate constants of donor–acceptor contact formation (*k*_+_) and dissociation (*k*_–_)

System	*D*_t_ (μm^2^ s^–1^)	*τ* C obs (μs)	*k*_+_ (ms^–1^)	*k*_–_ (ms^–1^)
*c-MYC*-(A)	221	49	10.6 ± 1.1	9.8 ± 1
*c-MYC*-(A) + K^+^	296	9	37.0 ± 4	74.0 ± 7
*c-MYC*-(A) + K^+^ + **BTC**	318	5	97.4 ± 10	102.6 ± 11
*c-MYC*-(A) + **BTC**	318	6	88.0 ± 9	78.6 ± 8
*h-TELO*-(A)	242	29	10.8 ± 1.1	23.7 ± 2.5
*h-TELO*-(A) + K^+^	283	9	41.7 ± 4.3	69.4 ± 7
*h-TELO*-(A) + K^+^ + **BTC**	314	7	71.4 ± 7.4	71.5 ± 7
*h-TELO*-(A) + **BTC**	318	7	66.8 ± 7	76.1 ± 8
	*D*_t_, *τ*Cobs = ±10%			

Next, the kinetic parameters for the end-to-end contact formation (*k*_+_) and dissociation (*k*_–_) of the K^+^-free and K^+^-folded quadruplexes are calculated (Fig. 3b and d, Tables 1 and S4, ESI†). The end-to-end contact formation of K^+^-free DNA corresponds to the initial intra-chain contact formation during the G-quadruplex formation (Fig. 3e).^42^ In contrast, the *k*_+_/*k*_–_ values of the folded quadruplexes indicate the motion of flanking sequences and the dye linkers (Fig. 3f).^39^ The K^+^-free *c-MYC*-(A) and *h-TELO*-(A) exhibit nearly identical *k*_+_ values of ∼10 ms^–1^. The *k*_+_ values are increased ∼3–4 fold for the K^+^-folded *c-MYC*-(A) and *h-TELO*-(A) quadruplexes. However, the K^+^-free *c-MYC*-(A) and *h-TELO*-(A) sequences show *k*_–_ values of 9.8 ms^–1^ and 23.7 ms^–1^, respectively. The K^+^-folded *c-MYC*-(A) and *h-TELO*-(A) quadruplexes exhibit a 7-fold and a 3-fold increase in *k*_–_ values, respectively. Upon binding to the **BTC**, *c-MYC*-(A) displays an ∼8–10 fold increase and the *h-TELO*-(A) displays a ∼6.0 and a ∼3.0 fold increase in the *k*_+_ and *k*_–_ values, respectively. Interestingly, the *k*_+_/*k*_–_ values obtained for single labeled *c-MYC*-(B) and *h-TELO*-(B) are also comparable to those of *c-MYC*-(A) and *h-TELO*-(A) (Fig. S9, Table S6, ESI†).

We presumed that ligand-induced changes in diffusion parameters are associated with the conformation of the DNA. The K^+^-free DNA sequences have a large surface area, which causes increased hydration on the surface of the DNA. This creates a constraint in flexibility in K^+^-free DNA and consequently, the rates of intra-chain contact formation and dissociation (*k*_+_/*k*_–_) have lower values (Fig. 3e). However, the **BTC**-mediated folding of K^+^-free DNA sequences into compact globular quadruplexes causes dehydration due to lower availability of the hydration sites resulting in high *k*_+_ and *k*_–_ values.

### Fluorescence lifetime monitored dynamics of DNA conformation distribution

The influence of **BTC** on the conformational dynamics of *c-MYC* and *h-TELO* quadruplexes are further investigated by measuring the donor decay of single and dual labeled sequences (Fig. S10 and S11, ESI†). The rate constant of FRET (*k*_FRET_) between donor and acceptor can be expressed as a simple function of D–A distances (*R*_DA_):2
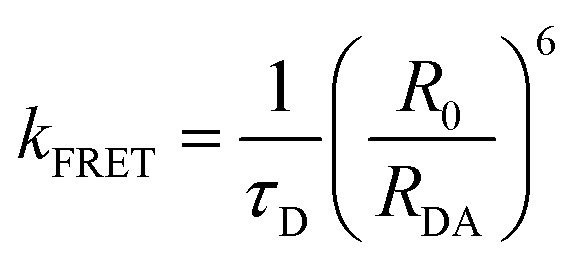
where, *R*_0_ represents the Förster distance and the *k*_FRET_ can be determined from the average lifetime (*τ*_avg_) of donor labeled *c-MYC*-(B) and *h-TELO*-(B) (*τ*_D_) and donor–acceptor labeled *c-MYC*-(A) and *h-TELO*-(A) (*τ*_DA_) (Tables S7 and S8, ESI†) using eqn (3).3
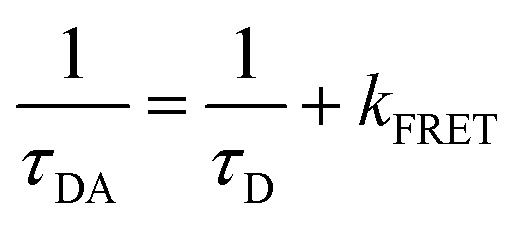



The *c-MYC*-(A) exhibits a *k*_FRET_ value of ∼0.8 ns^–1^ that increases to ∼1.3 ns^–1^ and ∼2.0 ns^–1^ in the presence of K^+^ and **BTC**, respectively. A similar increase in the *k*_FRET_ value is observed for *h-TELO*-(A) after the addition of K^+^ and **BTC**, respectively. For further illustration, the *R*_DA_ of *c-MYC*-(A) and *h-TELO*-(A) are estimated from the average FRET efficiency (*ε*_avg_) using eqn S21, ESI† (Tables S7 and S8, ESI†). The calculated *R*_DA_ is ∼49 Å for the *c-MYC*-(A), ∼44 Å for K^+^-folded *c-MYC*-(A), ∼42 Å for the **BTC** bound K^+^-folded *c-MYC*-(A) and ∼39 Å for the **BTC** folded *c-MYC*-(A) conformations. For *h-TELO*-(A), the *R*_DA_ value decreases from ∼55 Å in the K^+^-free-state to ∼44 Å in K^+^-folded state and it reduces further to 41 Å in the **BTC**-bound conformation. These distances are in close agreement with the sm-FRET data and demonstrate that the binding of **BTC** decreases the distance between the donor and acceptor labels, as compared to the K^+^-free and K^+^-folded *c-MYC*-(A) and *h-TELO*-(A) conformations. Together, these results indicate that **BTC** can template the formation of compact quadruplexes from the K^+^-free G-rich sequences that facilitate faster donor decay *via* donor-to-acceptor energy transfer.

### Analysis of ligand binding using NMR spectroscopy

**Fig. 4:**
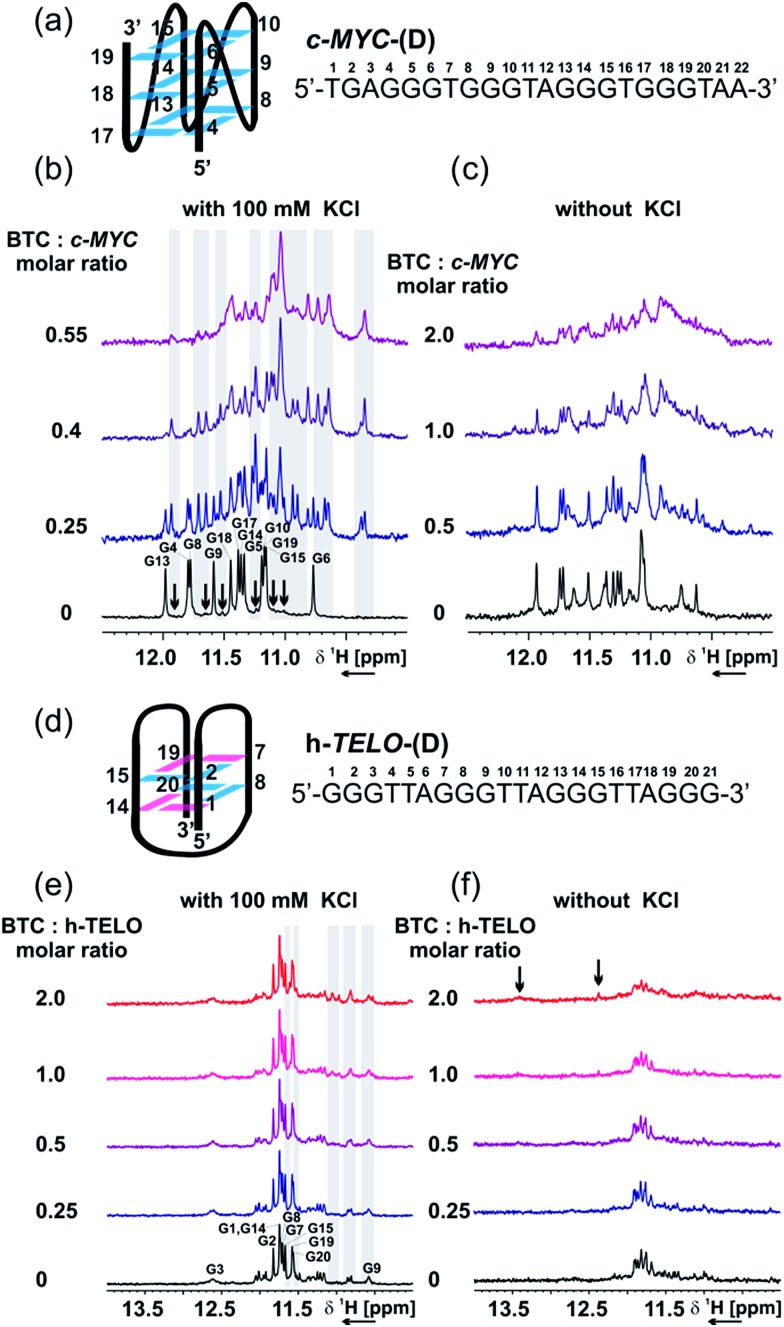
NMR titration of *c-MYC*-(D) and *h-TELO*-(D) in the presence and absence of **BTC** and K^+^. (a) Scheme of the major conformation of *c-MYC*-(D), as determined using NMR from Ambrus *et al.*^43^ [PDB code: ; 1XAV], with sequence and numbering. Imino region of the 1D ^1^H NMR spectrum of *c-MYC*-(D) in the presence of an increasing amount of **BTC**, with (b) or without (c) 100 mM KCl. (d) Scheme of the proposed structure of *h-TELO*-(D), with sequence and numbering. Imino region of the 1D ^1^H NMR spectrum of *h-TELO*-(D) in the presence of an increasing amount of **BTC**, with (e) or without (f) 100 mM KCl. Experimental conditions: 100 μM DNA, 25 mM Tris–HCl buffer (pH 7.4), 298 K, 600 MHz. Guanine residues in *anti* and *syn* conformations are represented in cyan and magenta, respectively.

NMR analysis showed that in the presence of K^+^, *c-MYC*-(D) exists as an equilibrium between a major parallel conformation,^43^ and one or more minor conformations (Fig. 4a), possibly featuring an all-parallel strand scaffold but different capping structures.^43^,^44^ Imino signals of the major conformation are labeled on the spectrum according to the numbering shown in Fig. 4a, while signals of the minor conformations are indicated with black arrows. In the absence of K^+^, the *c-MYC*-(D) sequence is partially pre-folded as a mixture of two or more conformations, as suggested by the signals detected in the imino region (Fig. 4c).

Addition of **BTC** to *c-MYC*-(D) shows that the ligand selectively interacts and stabilizes a minor conformation, both in the presence and absence of K^+^ (Fig. 4c and d). The signals of the imino protons of the major conformation of *c-MYC*-(D) are not perturbed by **BTC**. However, a set of signals, already detectable in the absence of **BTC** (minor conformation), becomes more intense upon the addition of ligand, which suggests that the ligand can interact with the *c-MYC*-(D) quadruplex *via* conformational selection. As suggested by the CD data (Fig. S14, ESI†), we propose that the minor conformation stabilized by **BTC** preserves the all-parallel scaffold of the major conformation (Fig. 4a), but presents different arrangements of the capping structures.

In K^+^ containing buffer, the ^1^H 1D imino pattern revealed that *h-TELO*-(D) exists predominantly as a 2-tetrad basket quadruplex, probably with looser capping structures compared to the structure reported by Lim *et al*.^17^ (Fig. 4d and e). Other minor conformations are also observed in the ^1^H NMR spectra of the *h-TELO*-(D) quadruplex. Addition of **BTC** to the *h-TELO* pre-folded in the presence of K^+^ induces weak chemical shift perturbations (highlighted with grey shadows, Fig. 4e) of the imino signals from the minor conformation and, possibly, from the guanine residues involved in the capping structure (G9 and G21). In the absence of K^+^, the *h-TELO*-(D) is found to be partially folded, as suggested by the imino signals detectable in the Hoogsteen hydrogen bond region (Fig. 4f).

Interestingly, upon addition of **BTC**, at a ratio [Ligand] : [DNA] = 2, a broad signal at 13.5 ppm, indicating the formation of a Watson/Crick (WC) base pair, and a sharp signal at 12.5 ppm, possibly arising from the carbazole NH group or from the newly formed Hoogsteen imino bonds between guanines, were detected. These findings suggest that in the absence of K^+^, **BTC** is able to promote the restructuring of the capping structures *via* formation of intra- or inter-loop WC-A : T interactions.

In contrast with that observed by sm-FRET (Fig. S4, ESI†), the aromatic region of the ^1^H NMR spectra of *h-TELO*-(D) (Fig. S14c and d, ESI†) reveals that in the absence of K^+^, **BTC** is able to promote G-quadruplex folding only partially. Taken together, the NMR and CD data support the single-molecule results, confirming that the ligand is able to stabilize the *c-MYC*-(D) and *h-TELO*-(D) G-quadruplexes. However, the details of the interaction process, particularly in the absence of K^+^, might be different to the one reported by single-molecule studies, due to the different experimental conditions. In particular, the DNA concentration used in the NMR studies is 10^6^-fold higher than the concentration used in the sm-FRET studies.

## Conclusions

In conclusion sm-FRET analysis reveals that the binding of **BTC** can shift the equilibria of K^+^-free *c-MYC* and *h-TELO* DNA sequences, which existed as ensembles of unfolded and partially folded quadruplex structures, to a single folded conformation. FCS studies demonstrate that **BTC** alters the rod shaped DNA sequences into globular quadruplexes with a decrease in hydrodynamic radii. NMR analysis shows that **BTC** binds to a minor conformation of *c-MYC* by conformational selection, and is capable of promoting a restructuring of the loop–loop interactions in the basket conformation formed by *h-TELO*. These studies provide quantitative analysis of small molecule-mediated dynamic conformational changes of human G-quadruplexes that would be applicable for a range of interacting ligands with a variety of quadruplex forming sequences.

## Supplementary Material

Supplementary information
