# Supplementary material for: Small molecule regulated dynamic structural changes of human G-quadruplexes
Source: Chem Sci. 2016 Feb 12;7(5):3279–85. doi: 10.1039/c6sc00057f (PMC6006475; doi:10.1039/c6sc00057f)
Supplement: Supplementary file 1 [file SC-007-C6SC00057F-s001.pdf]

Electronic Supplementary Material (ESI) for Chemical Science.
This journal is © The Royal Society of Chemistry 2016

SUPPORTING INFORMATION

Small Molecule Regulated Dynamic Structural Changes of Human G-quadruplexes

Manish Debnath,[a] *Shirsendu Ghosh,*[b] *Deepanjan Panda,* [a] *Irene Bessi,*[c] *Harald Schwalbe,*[c] *Kankan Bhattacharyya,* [b] *Jyotirmayee Dash*[a]*

[a] Department of Organic Chemistry, Indian Association for the Cultivation of Science, Jadavpur, Kolkata-700032, India; email:ocjd@iacs.res.in

[b] Department of Physical Chemistry, Indian Association for the Cultivation of Science, Jadavpur, Kolkata-700032, India

[c] Institute of Organic Chemistry and Chemical Biology, Goethe University Frankfurt and Centre for Biomolecular, Magnetic Resonance, Max-von-Laue Strasse 7, 60438, Frankfurt am Main, Germany

Table of contents

**1.0**	**General information**	**S3**
**2.0**	**Synthesis of bis-triazolylcarbazole ligand (BTC)**	**S4**
**3.0**	**NMR spectra of carbazole derivatives**	**S6**
**4.0**	**FRET Melting analysis**	**S8**
**5.0**	**Confocal microscopy**	**S9**
**6.0**	**Donor shot noise data**	**S11**
**7.0**	**sm-FRET analysis**	**S13**
**8.0**	**FCS analysis**	**S15**
**9.0**	**Lifetime data**	**S22**
**10.0**	**CD spectroscopy**	**S24**
**11.0**	**NMR spectroscopy**	**S26**

1.0 General information:

All solvents and reagents were purified by standard techniques reported in Armarego, W. L. F., Chai, C. L. L., Purification of Laboratory Chemicals, 5th edition, Elsevier, 2003; or used as supplied from commercial sources (Sigma-Aldrich Corporation® unless stated otherwise). All reactions were generally carried out under inert atmosphere unless otherwise noted. TLC was performed on Merck Kieselgel 60 F254 plates, and spots were visualized under UV light. Products were purified by flash chromatography on silica gel (100-200 mesh, Merck). ^{1}H and ^{13}C NMR spectra were recorded on either Brüker ADVANCE 500 (500MHz and 125 MHz), or JEOL 400 (400 MHz and 100 MHz) instruments using deuterated solvents as detailed and at ambient probe temperature (300 K). Chemical shifts are reported in parts per million (ppm) and referred to the residual solvent peak. The following notations are used: singlet (s); doublet (d); triplet (t); quartet (q); multiplet (m); broad (br). Coupling constants are quoted in Hertz and are denoted as *J*. Mass spectra were recorded on a Micromass® Q-Tof (ESI) spectrometer. All DNA oligonucleotides were purchased from Sigma-Aldrich. The melting point (T_m) of labeled and unlabeled *c-MYC* was 70.2 and the melting point of labeled and unlabeled *h-TELO* was 55.3 as supplied by the manufacturer.

2.0 Synthesis of bis-triazolylcarbazole ligand (BTC):

Sonogashira coupling of diiodo-carbazole **S1** with 2-methylbut-3-yn-2-ol **S2** followed by removal of the acetone group afforded the dialkyne **1** in 92% overall yield for the two steps. Carbazole dialkyne **1** was treated with the azide **2** using catalytic $CuSO_4$, sodium ascorbate in *t*-BuOH/H_2O (3:1)[1] under microwave condition at 70 °C for 4 h to give the corresponding bis-triazolylcarbazole derivative **BTC** in 73% yield (**Figure S1**).

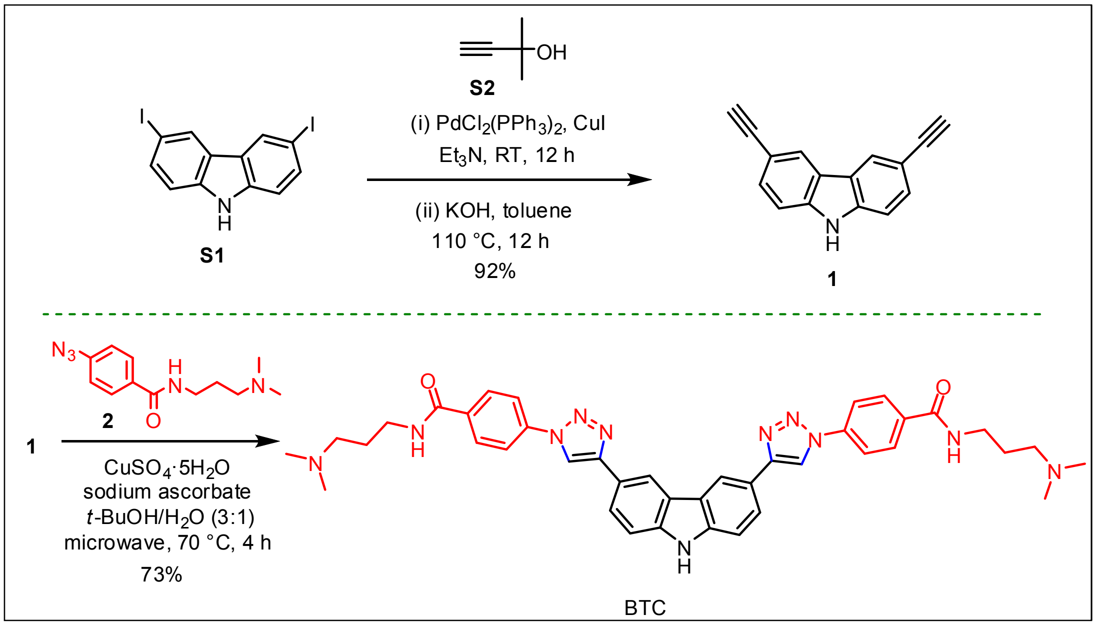

Figure S1. Modular synthesis of BTC ligand.

Preparation of 3,6-diethynyl-9H-carbazole 1: To a solution of 3,6-diiodocarbazole (250 mg, 0.597 mmol) in Et_3N (5 mL) added $PdCl_2(PPh_3)_2$ (41.9 mg, 0.0597 mmol) and CuI (11.4 mg, 0.0597 mmol) at room temperature. The mixture was stirred for 30 min and then 2-methylbut-3-yn-2-ol **4** (0.347 mL, 3.582 mmol) was added drop-wise. The resulting mixture was stirred under an argon atmosphere for 12 h, concentrated, washed with brine and dried over anhydrous Na_2SO_4. The crude product was purified by column chromatography.

[1] V. V. Rostovtsev, L. G. Green, V. V. Fokin, K. B. Sharpless, *Angew.Chem. Int. Ed.* 2002, **41**, 2596-2599.

Then the resulting alcohol was refluxed with 5 equiv. KOH in toluene under an argon atmosphere for 12 h. The reaction mixture was concentrated, washed with brine and dried over anhydrous Na_2SO_4.The crude product was purified by column chromatography to give the dialkyne **1** (118.22 mg, 92% yield) as a yellow solid. ^{1}H NMR (500 MHz, $CDCl_3$): 8.26 (s, 1H), 8.20 (s, 2H), 7.57 (d, 2H, J = 8.2 Hz), 7.36 (d, 2H, J = 8.2 Hz), 3.08 (s, 2H). ^{13}C NMR (100 MHz, $CDCl_3$): 139.8, 130.5, 124.9, 122.9, 113.6, 110.9, 84.7, 75.6.

Preparation of BTC: A mixture of the dialkyne carbazole **1** (100 mg, 0.465 mmol), the azide **2**, $CuSO_4{\cdot}5H_2O$ (11.6 mg, 0.0465 mmol) and sodium ascorbate (9.2 mg, 0.0465 mmol) were taken in 3mL ^{t}BuOH/H_2O (3:1). The mixture was then heated for 4 h at 70 °C under microwave irradiation. The reaction mixture was cooled down to room temperature and the solvents were concentrated. The crude product was purified by flash column chromatography [CH_2Cl_2 (100%) to CH_2Cl_2/MeOH (10:1) to CH_2Cl_2/MeOH/NH_4OH (10:1:0.5)] to give **BTC** (241 mg, 73%) as a yellow solid. ^{1}H NMR (DMSO-d^6, 500 MHz): 11.61 (s, 1H), 9.42 (s, 2H), 8.81 (s, 2H), 8.72 (s, 2H), 8.12 (s, 8H), 8.04 (d, 2H, J = 8.4 Hz), 7.65 (d, 2H, J = 8.4 Hz), 3.32 (4H, merged with water peak), 2.48 (s, 4H), 2.30 (s, 12H), 1.75 (t, 4H, J = 6.7 Hz); ^{13}C NMR: (125 MHz, DMSO-d^6): 165, 148.7, 140.2, 138.4, 134.2, 128.9, 123.8, 122.8, 121.1, 119.3, 118.5, 117.3, 111.7, 56.3, 44.4, 37.5, 26.4. HRMS (ESI) calculated for [$C_{40}H_{44}N_{11}O_2$ $(M+H)^+$]: 710.3679, Found 710.3740.

3.0 NMR spectra of carbazole derivatives:

[1]H Spectra of 3,6-diethynyl-9H-carbazole 1:

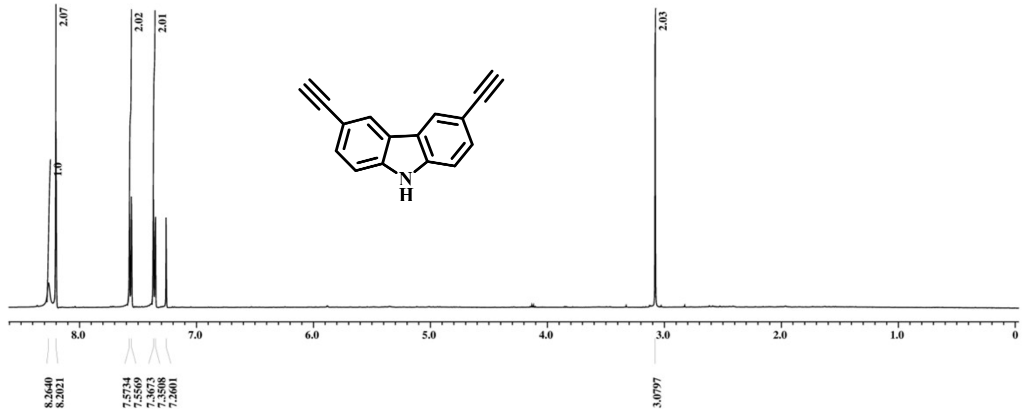

[13]C Spectra of 3,6-diethynyl-9H-carbazole 1:

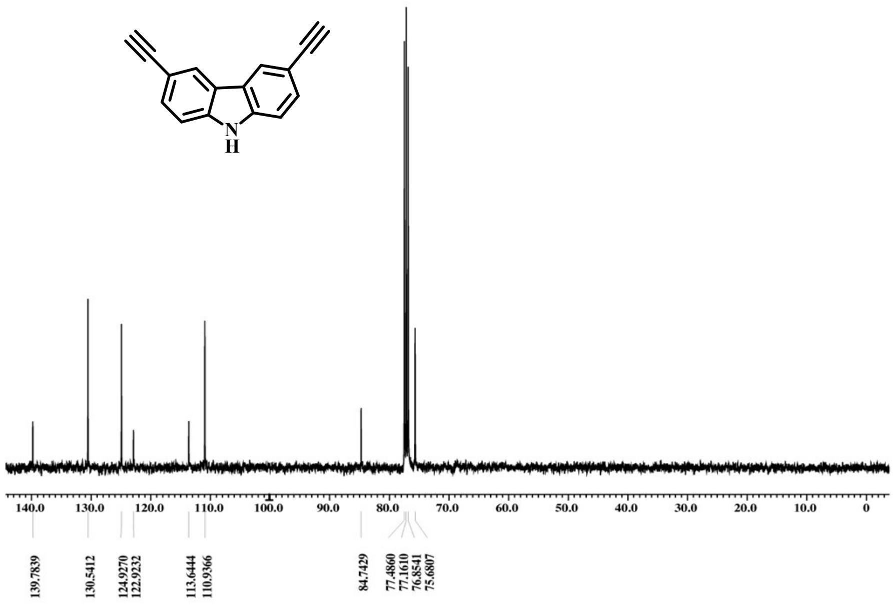

^{1}H Spectra of BTC:

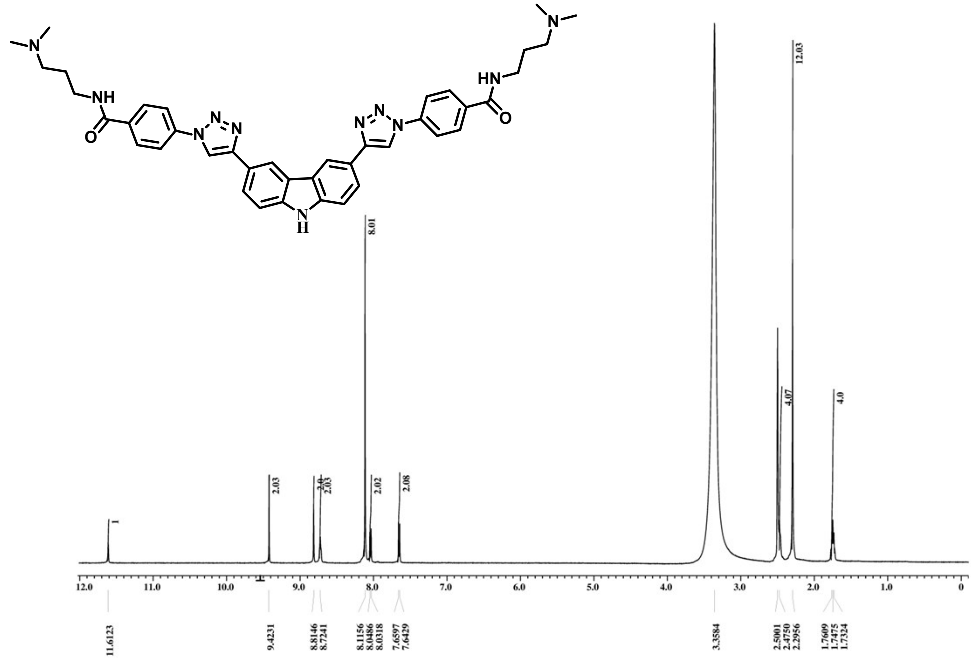

^{13}C Spectra of BTC:

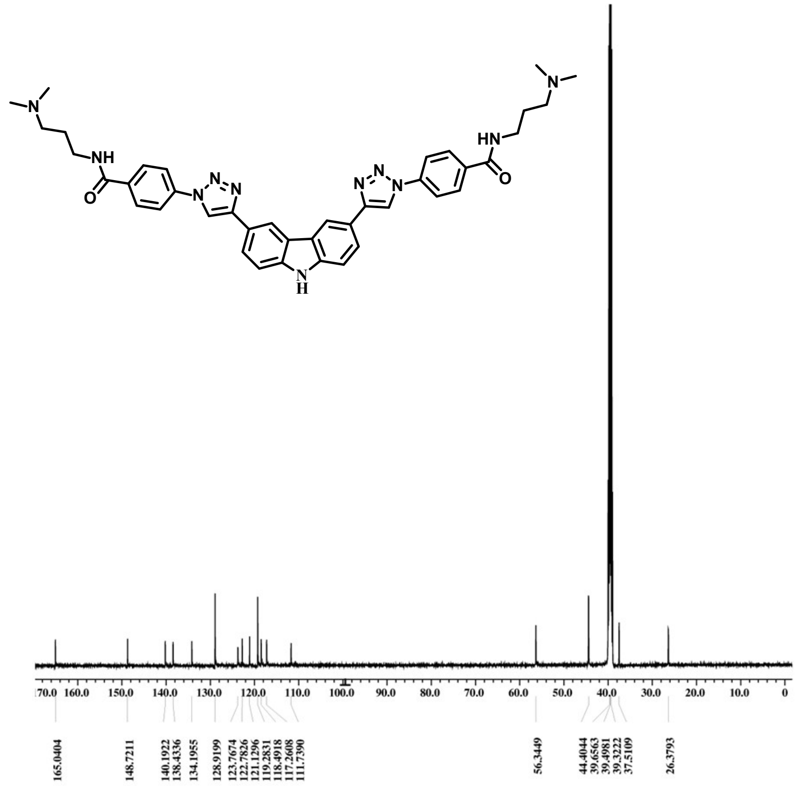

4.0 FRET Melting analysis:

Stock solutions having 200 µM concentration of **BTC** was prepared in MQ water. Dual-labeled *c-MYC*-(A) and *h-TELO*-(A) sequences and a duplex hairpin DNA (*ds* DNA) sequence were diluted in 50 mM potassium cacodylate buffer (pH 7.4). For the experiments in absence of K^+, the dual-labeled oligonucleotides were diluted in MQ water (pH ~ 7). Dual-labeled DNA was annealed at a concentration of 200 nM by heating at 95 ºC for 5 min followed by cooling to room temperature. The 96-well plates were prepared by aliquoting 50 µL of the annealed DNA into each well, followed by 50 µL of **BTC** at different concentrations (0.05, 0.1, 0.2, 0.4, 0.7, 1.0 µM). Measurements were made in triplicate with an excitation wavelength of 483 nm and a detection wavelength of 533 nm using a LightCycler® 480-II System RT-PCR machine (Roche). Final analysis of the data was carried out using Origin Pro 8 data analysis.

Sequences used in this study:

c-MYC-(A): 5′-FAM-d($TGAG_3TG_3TAG_3TG_3TA_2$)-TAMRA-3′

h-TELO-(A): 5′-FAM-d($G_3TTAG_3TTAG_3TTAG_3$)-TAMRA-3′

*ds*DNA: 5′-FAM-d(TAT AGC TAT A HEG TAT AGC TAT A)-TAMRA-3′

Table S1. FRET stabilization potential (ΔT_m) values of *c-MYC*-(A) and *h-TELO*-(A) with increasing concentration of **BTC** in the presence and absence of K^+.

[**BTC**] (µM)	ΔT_m of DNA sequences (° C)					
	c-MYC-(A)[a]	*h-TELO*-(A)[a]	*c-MYC*-(A) + K^{+a}	*h-TELO*-(A) + K^{+a}	*ds* DNA	*ds* DNA+ K^+
0	0	0	0	0	0	0
0.05	5.8	4.5	9.9	3.8	0.2	0.2
0.1	11.9	9.2	16.7	8.6	0.4	0.5
***0.2**	**23.5**	19.9	**24.0**	17.8	0.9	1
***0.4**	24.0	**38.5**	24.5	**38.7**	1.4	1.5
0.7	24.1	38.8	24.6	38.9	1.9	2
1	24.2	39.0	24.6	39.1	2.9	3
[a]SD = ΔT_m± 5%						

The concentrations of **BTC to reach saturation were in the nanomolar range; ~ 200 nM for *c-MYC*-(A) and ~ 400 nM for *h-TELO*-(A) quadruplex.

5.0 Confocal microscopy:

Single molecule FRET: For sm-FRET studies, very dilute solution of dual labeled *c-MYC*-(A) and *h-TELO*-(A) DNA sequences (~ 100 pM) in 10 mM Tris·HCl (pH 7.4) containing 100 mM KCl and MQ water, pH 7.4 were used. **BTC** was added in the final concentration of 1 equivalent (equiv.) of the DNA used i.e., 100 pM in case of the sm-FRET. We used the PicoQuant, Micro-Time 200 confocal setup with an inverted optical microscope (Olympus IX-71).[2] We used a pulsed diode laser with a repetition rate of ~ 40 MHz. We have kept the laser power at or below ~50 μW. Fluorescence from the DNA labeled with the dye was separated with a dichroic mirror (HQ490DCXR, Chroma). To block the exciting laser light, a suitable long-pass filter (510LP, Chroma, for excitation at 470nm) was used before the detectors. The fluorescence was focused through a pinhole (30 μm). For fluorescence cross-correlation and sm-FRET studies, the donor (FAM) and the acceptor (TAMRA) fluorescence signal were captured separately using a dichroic mirror (540DCLP) and two single photon avalanche diodes (SPADs). Two additional band pass filters (FF01-520/35 for the donor and HQ580/35 for the acceptor) were used to further separate the donor and acceptor fluorescence.

In FRET experiments for the freely diffusing system, molecules labeled with donor (FAM) and acceptor (TAMRA) pass through the confocal volume on a bare slide and the donor dye is excited by laser illumination. Fluorescence bursts of both donor and acceptor (excited via FRET) are then observed and from this, efficiency of FRET may be calculated. For the freely diffusing DNA, FRET analysis is, however, limited by the transit time (binning time typically ~ 2.5 ms) through observation volume of dimension 235 nm [0.61λ/numerical aperture, NA = 1.2, λ = 470 nm].The total time collected to generate a single histogram was ~15 min. The background count was found to be ~ 500 counts/s and the average count for a burst was ~2500 counts/s. In the MCS plots a background of 500 counts/s is subtracted from the data to obtain background corrected images (**Figure 2 and Figure S4**). The average signal strength for a burst indicates approximately 5X signal strength of background. However we have observed bursts having maximum signal strength ~ 4000 counts/s, which indicates approximately 8X signal strength of background.

Efficiency of FRET (ε_{FRET}) is given by,[3]

---

[2] S. Ghosh, C. Ghosh, S. Nandi, K. Bhattacharyya, *PhysChemChemPhys.* 2015, **17**, 8017-8027.

[3] J. R. Lakowicz, Principles of fluorescence spectroscopy; 3rd ed.; Springer: New York, (2006).

$$\varepsilon_{FRET} = \frac{I_A}{\gamma I_D + I_A} \text{.............(S1)}$$

Where, I_A and I_D are the background and cross-talk corrected acceptor and donor intensities. The correction factor γ may be expressed as,

$$\gamma = \frac{\varphi_A \eta_A}{\varphi_D \eta_D} \text{...........(S2)}$$

The value of γ is obtained from the quantum yield of donor (φ_D) and acceptor (φ_A) and the calibration curves for the detection efficiency of donor (η_D) and acceptor (η_A) SPADs. The value of γ was determined for each system separately considering change in quantum yields of both donor and acceptor labeled DNA in the presence and absence of **BTC** and K^+.

The distance between the dye pairs (R_{DA}) is calculated from each FRET efficiency value from the relation,

$$R_{DA} = R_0 \left[\frac{1 - E}{E} \right]^{\frac{1}{6}} \text{.............(S3)}$$

R_0 (Förster distance) was determined from the spectral overlap, *J*(λ), between the donor emission and the acceptor absorption as follows,

$$R_0 = 0.211 \left[\kappa^2 \eta^{-4} Q_D J(\lambda) \right]^{1/6} \text{...........}(S4)$$

$$J(\lambda) = \frac{\int_0^\infty F_D(\lambda) \varepsilon_A(\lambda) \lambda^4 d\lambda}{\int_0^\infty F_D(\lambda) d\lambda} \text{...........}(S5)$$

R_0 (Förster distance) for the FAM-TAMRA pair of fluorescent dye is 55 Å (assuming a rotational diffusion randomized to the dipole orientation factor κ^2 of 2/3)[4].

[4] R. M. Clegg, *Methods Enzymol.* 1992, **211**, 353-388.

6.0 Donor shot noise data:

The sm-FRET experiments are often complicated by shot noise.[5,6] The contribution of shot noise in each FRET peak were calculated[7].

Table S2. FRET peak widths for folded and unfolded G-quadruplex DNA.

System	Peak efficiency	σ_{sn}	σ_m	σ_m / σ_{sn}
c-MYC	0.42	0.14	0.16	1.14
	0.6	0.14	0.21	1.47
	0.82	0.11	0.06	**0.54**
c-MYC + K^+	0.56	0.14	0.05	**0.36**
	0.78	0.12	0.18	1.5
c-MYC + **BTC**	0.85	0.10	0.13	1.3
c-MYC + **BTC** + K^+	0.83	0.09	0.13	1.44
h-TELO	0.51	0.16	0.25	1.56
h-TELO + K^+	0.75	0.11	0.18	1.64
h-TELO + **BTC**+ K^+	0.85	0.10	0.14	1.4
h-TELO + **BTC**	0.88	0.11	0.14	1.27

The width of the FRET efficiency peak from shot noise, σ_{sn}, is given by,

$$\sigma_{sn} = \sqrt{\langle E_m \rangle (1 - \langle E_m \rangle) \langle N^{-1} \rangle} \text{............} (S6)$$

where, $\langle N^{-1} \rangle$ is the mean of the reciprocal number of the donor and acceptor photons and E_m is the measured FRET efficiency. The mean is calculated using the bursts that contribute to the FRET efficiency peak of the labelled DNA. The contribution of shot noise was found to be significantly higher in the FRET peak at ~ 0.82 and ~ 0.56 in free and K^+ folded *c-MYC* respectively. Hence we didn't assign these two peaks as sub-populations of DNA secondary structures.

---

[5] E. Nir, X. Michalet, K. M. Hamadani, T. A. Laurence, D. Neuhauser, Y. Kovchegov, S. Weiss, *J. Phys. Chem. B* 2006, **110**, 22103-22124.

[6] M. Antonik, S. Felekyan, A. Gaiduk, C. A. M. Seidel, *J. Phys. Chem. B* 2006, **110**, 6970-6978.

[7] K. A. Merchant, R. B. Best, J. M. Louis, I. V. Gopich and W. A. Eaton, *Proc. Natl. Acad. Sci. U. S. A.*, 2007, **104**, 1528-1533.

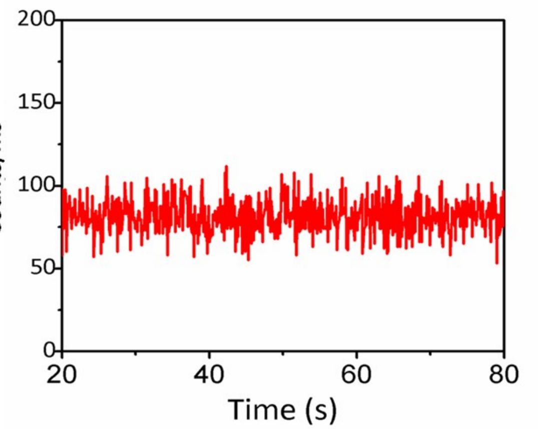

Figure S2. Donor [*c-MYC*-(B)] mcs trace showing no appreciable bleaching of emission of donor with time.

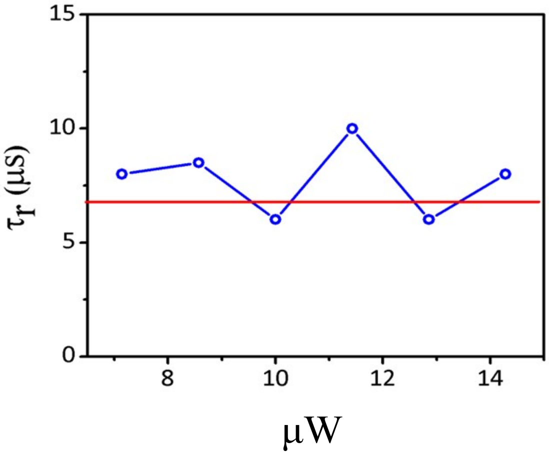

Figure S3. The relaxation times of the only donor labeled *c-MYC*-(B) as a function of laser power (μW).

7.0 sm-FRET analysis:

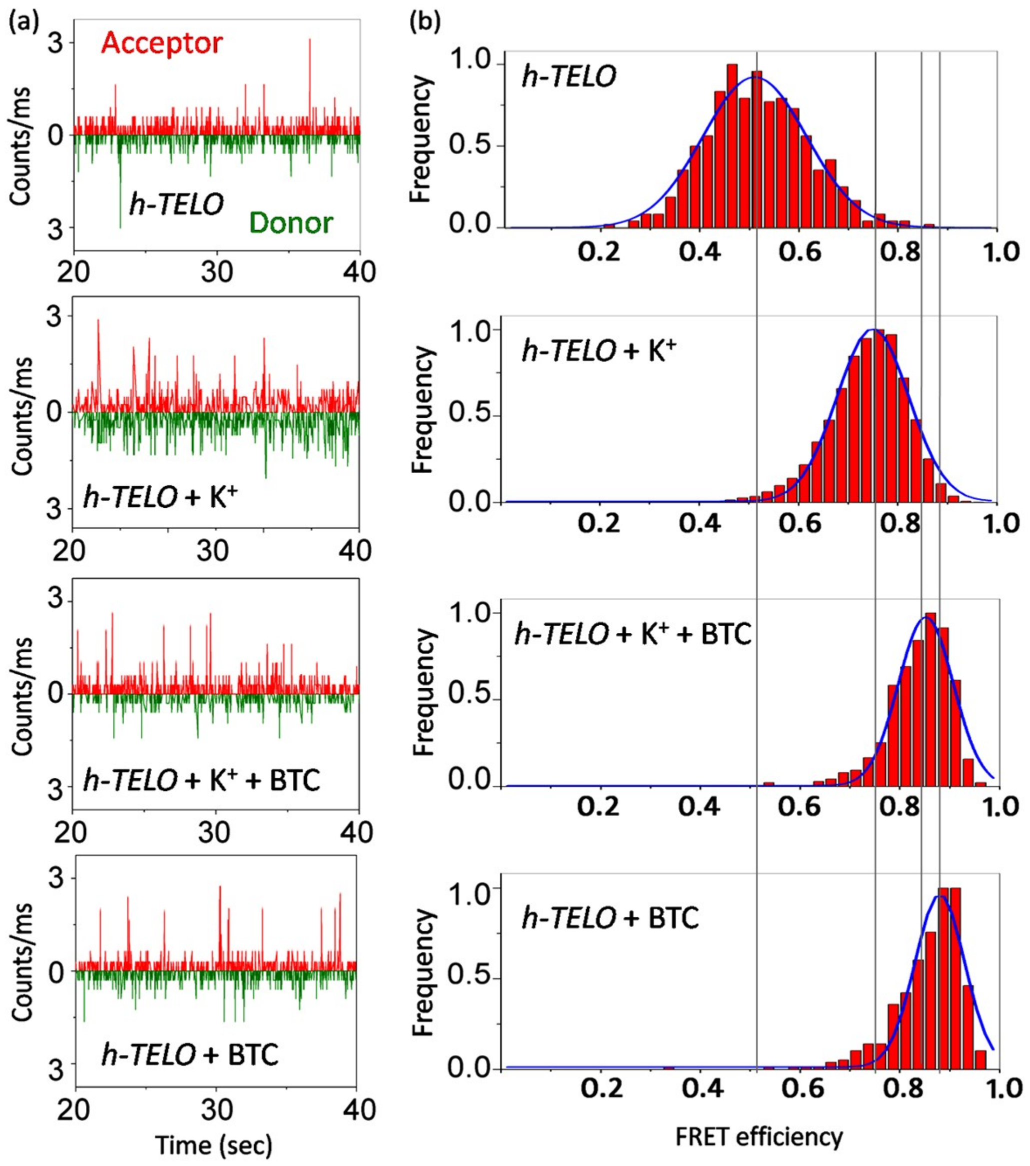

Figure S4. The sm-FRET analysis of *h-TELO*-(A) in the presence and absence of **BTC** and K^+. Photon bursts of donor/acceptor (background corrected) (a), and FRET efficiency distributions (b) of 100 pM dual fluorescently labeled *h-TELO*-(A) G-quadruplex forming sequence in the presence and absence of K^+ and **BTC.**

The fits were determined by best fit method as we tried to obtain best fit by fitting the histograms into least number of distributions. We started fitting from single Gaussian distribution, then two and three Gaussian distribution. The first histogram for *c-MYC*-(A) was fit to three histograms. We have tried to fit it into two Gaussian distribution. But it can be readily seen from the data provided in the **Figure S5** that the histogram is only better fitted in three Gaussian distribution. The second histogram of *c-MYC*-(A) + K^+ is however fitted into a single Gaussian distribution in the **Figure 2**. The rest of two histograms are fitted with single Gaussian distribution. All the histograms of *h-TELO*-(A) were easily fitted into single Gaussian distribution.

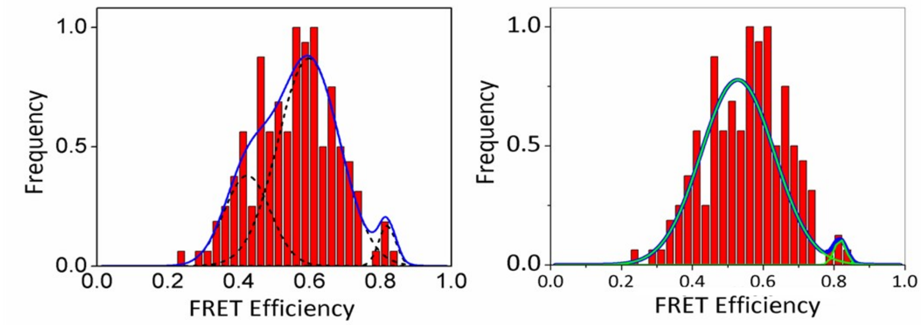

Figure S5. The FRET histograms of *c-MYC*-(A) fitted with tri-exponential function (Left) and fitted with bi-exponential function (Right). It can be clearly seen that the histogram is better fitted with tri-exponential function.

Table S3. The FRET efficiency (%) and corresponding donor-acceptor distances (R_{DA}).

System	Efficiency (%)	R_{DA}(Å)
c-MYC-(A)	0.41 (30)	58.43
	0.59 (68)	51.76
	0.82(02)	42.72
c-MYC-(A) + K^+	0.56 (03)	52.83
	0.78 (97)	44.54
c-MYC-(A) + K^+ + **BTC**	0.83 (100)	42.23
c-MYC-(A) + **BTC**	0.85 (100)	41.19
h-TELO- (A)	0.51 (100)	54.63
h-TELO-(A) + K^+	0.76 (100)	45.3
h-TELO-(A) + K^+ + **BTC**	0.86 (100)	40.6
h-TELO-(A) + **BTC**	0.88 (100)	39.45

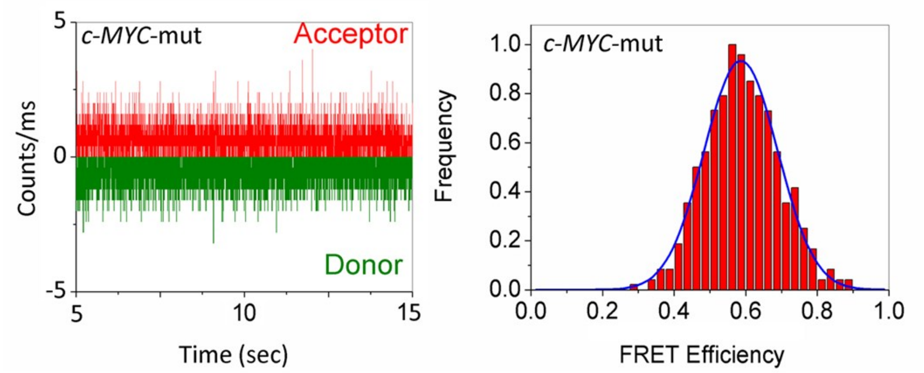

Figure S6. Photon bursts of donor/acceptor (left), and FRET efficiency distributions (right) of 100 pM dual labeled *c-MYC*-mut (*c-MYC*-mut = FAM-5′-TGAAATTGAATTGAATGAATAA-3′-TAMRA).

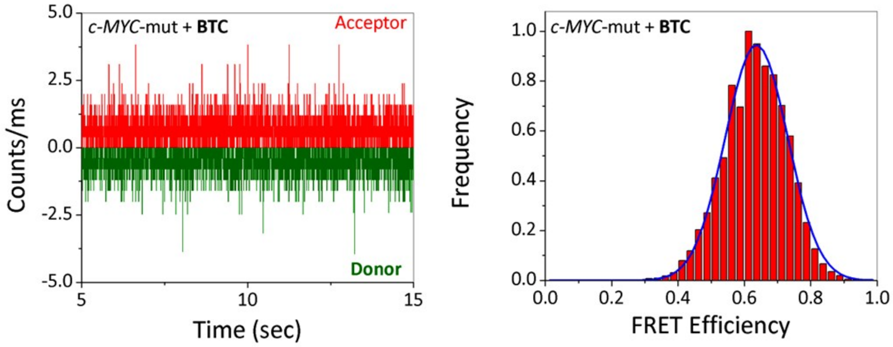

Figure S7. Photon bursts of donor/acceptor (left), and FRET efficiency distributions (right) of 100 pM dual labeled *c-MYC*-mut in the presence of **BTC**; (*c-MYC*-mut = FAM-5′-TGAAATTGAATTGAATGAATAA-3′-TAMRA).

8.0 FCS analysis:

FCS measures the time-dependent fluctuations in fluorescence intensity of a few reporter molecules present in the small observation volume. The fluctuations in the fluorescence intensity may result from the diffusion of the labeled molecule in and out of the focus or during the conformational change. Fluorescence intensity fluctuations are quantified by accumulating their autocorrelation function, which provides measurement of intramolecular dynamics of a reporter molecule. The interaction between the donor and acceptor dyes of the dual labeled oligonucleotide sequences can produce fluctuation in both open chain and folded conformation with the characteristic rates of donor acceptor contact formation k_+ and dissociation k_-. These fluctuations were measured by accumulating the autocorrelation function of the fluorescent light (FCS). The autocorrelation function consists of contribution from the molecular diffusion and the chemical kinetics. The molecular diffusion time reveals the information about the change in molecular size and the kinetics data reflects the rate of end-to-end contact formation due to the conformational change in DNA structure.

To elucidate the diffusional properties and kinetics of end-to-end contact formation of free and ligand-G-quadruplex DNA complex, we measured the FCS curve of *c-MYC*-(A) and *h-TELO*-(A) in the presence and absence of **BTC** (1 equivalent i.e. 10 nM in case of FCS) and 100 mM KCl. A dilute solution (~10 nM) of the dual labeled DNA was used for FCS measurement. The total time window for collecting FRET-FCS was ~ 30 min. Fluorescence fluctuations caused by diffusion or conformational change between folded and unfolded state can be expressed by the autocorrelation function G(τ),[8]

$$G(\tau)=\frac{1}{N}\left[1+\frac{\tau}{\tau_D}\right]^{-1}\left[1+K^{C}_{obs}\times \exp(-\frac{\tau}{\tau^{C}_{obs}})\right] \text{........(S7)}$$

Where N is the average number of labeled DNA in the observed volume, τ_D is the molecular diffusion time, K^{C}_{obs} is the observed amplitude of the intra-chain contact formation kinetics due to changes of the donor-acceptor distance, and τ^{C}_{obs} is the observed time of intra-chain contact formation. τ_D can be expressed in terms of diffusion coefficient (D_t) as,

$$\tau_D=\frac{r^2}{4D_t} \text{............... } (S8)$$

Where, r is the transverse radius.

The diffusion coefficient is inversely proportional to the frictional coefficient and hydrodynamic radius as described by the Stokes-Einstein equation for the spherical objects,[3]

$$D_t=\frac{k_B T}{f} \text{...................}(S9)$$

$$D_t=\frac{k_B T}{6\pi\eta R_h} \text{.................}(S10)$$

Where, k_B is the Boltzmann constant, T is the temperature in Kelvin, η is the viscosity of the solvent used and R_h is the hydrodynamic radius of the diffusing object. The frictional coefficient of a cylindrical or rod shaped object is higher than that of a sphere having the same volume. The frictional coefficient of the rod shaped object is described as,[9]

---

[8] J. Choi, S. Kim, T. Tachikawa, M. Fujitsuka and T. Majima, *J. Am. Chem. Soc.* 2011, **133**, 16146-16153.

[9] A. Ortega, J. García de la Torre, *J. Phys. Chem. B* 2003, **119**, 9914-9919.

$$f = f_0 \frac{(2/3)^{1/3} P^{2/3}}{\ln(2P) - 0.30} \text{.........................}(S11)$$

Where, P is the ratio of half length and radius of the cylinder. The average length and radius were roughly calculated for the single stranded *c-MYC*-(A) to be 7.0 nm and 0.3 nm. The average length and radius were roughly 6.7 nm and 0.3 nm for *h-TELO*-(A). f_0 is the frictional coefficient for the sphere having same volume as the rod having frictional coefficient f. The ratio of the frictional coefficient of the rod shaped free DNA and **BTC** bound K^+ folded spherical quadruplex directly corresponds to the ratio of hydrodynamic radius.

From the observed time and observed amplitude of end-to-end contact formation the rate constant for donor-acceptor contact formation (k_+) and dissociation (k_-) can be determined by using following relation:[10]

$$K_{obs}^{C} = \frac{k_+}{k_-} \text{..................}(S12)$$

and,
$$\tau_{obs}^{C} = \frac{1}{k_+ + k_-} \text{............}(S13)$$

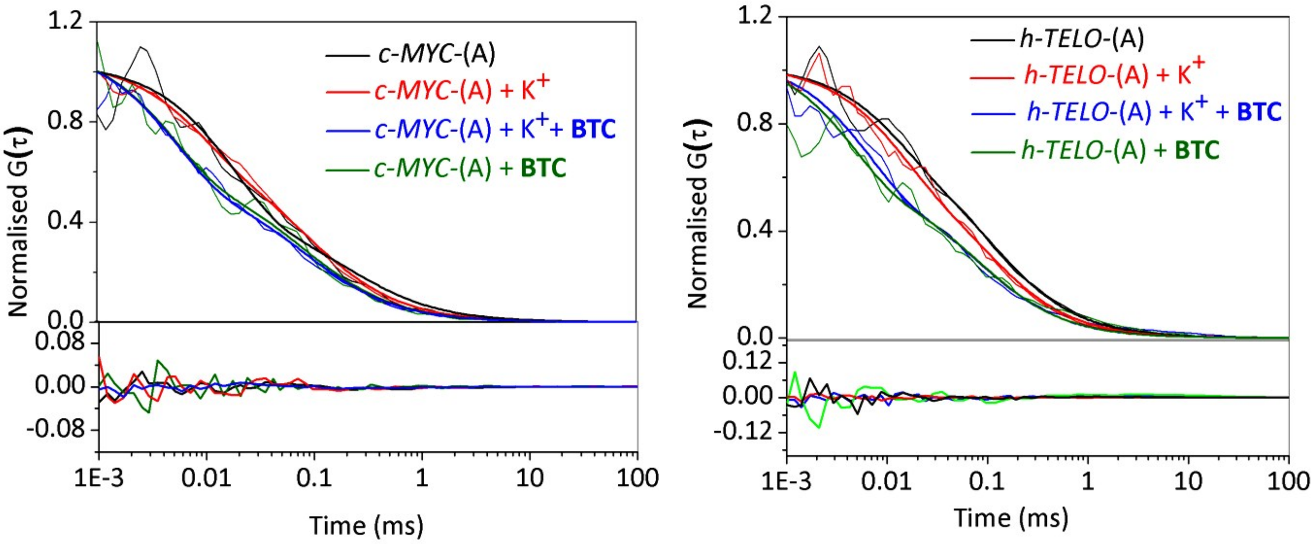

---

[10] J. Choi, S. Kim, T. Tachikawa, M. Fujitsuka, T. Majima, *J. Am. Chem. Soc.* 2011, **133**, 16146-16153.

Figure S8. Representative fitting result of FCS data of *c-MYC*-(A) and *h-TELO*-(A) in the presence and absence of K^+ and **BTC.** Lower panel is the residual of the fitting result.

Table S4. Table representing diffusion coefficient (D_t), diffusion time (τ_D), rate constants of donor acceptor contact formation (k_+), dissociation ($k\_$) of *c-MYC*-(A) and *h-TELO*-(A), the observed amplitude of the donor acceptor contact formation kinetics due to changes of the donor-acceptor distance ($\mathbf{K^C_{obs}}$), and the observed time of donor acceptor contact formation $\boldsymbol{\tau^C_{obs}}$ using equation 1.

System	τ_D (μs)[a]	D_t (μm²/s)[a]	K^C_{obs}[a]	τ^C_{obs} (μs)[a]	k_+(ms⁻¹)	k_ (ms⁻¹)
c-MYC-(A)	115	221	1.077	49	10.6 ± 1.1	9.8± 1
c-MYC-(A) + K^+	86	296	0.5	9	37.0 ± 4	74.0±7
c-MYC-(A) + K^+ + **BTC**	80	318	0.95	5	97.4 ± 10	102.6 ± 11
c-MYC-(A) + **BTC** (1 eq)	80	318	1.12	6	88.0 ± 9	78.6 ± 8
h-TELO-(A)	105	242	0.46	29	10.8 ± 1.1	23.7 ± 2.5
h-TELO-(A) + K^+	90	283	0.6	9	41.7 ± 4.3	69.4 ± 7
h-TELO-(A) + K^+ + **BTC**	81	314	0.99	7	71.4 ± 7.4	71.5 ± 7
h-TELO-(A) + **BTC** (1 eq)	80	318	0.88	7	66.8 ± 7	76.1 ± 8

[a] ±10 %, calculated from the standard deviation of the fitting of each data set.

For FCS studies with single donor labeled samples, we have used dilute solution (~ 10 nM) of the FAM labeled DNA. The number of molecules within the confocal volume is found to be ~ 10.

The autocorrelation function G(τ) of fluorescence intensity is defined as[11]

$$G(\tau)=\frac{<\delta F(0)\delta F(\tau)>}{<F>^2} \quad \text{(S14)}$$

Where <F> is the average intensity and δF (τ) is the fluctuation in intensity at a delay time τ around the mean value, i.e., δF (τ) = <F> - F(τ).

We have fitted the FCS traces to single component diffusion and one component relaxation model. For this model, the autocorrelation function is given by,[12]

$$G(\tau)=\frac{1-F+F\exp(-\tau/\tau_R)}{N(1-F)}(1+\frac{\tau}{\tau_D})^{-1}(1+\frac{\tau}{\omega^2\tau_D})^{-1/2}$$

[11] Z. Petrášek, P. Schwille, *Biophys. J.* 2008, **94**, 1437-1448.

[12] K. Chattopadhyay, S. Saffarian, E. L. Elson, C. Frieden, *Biophys. J.* 2005, **88**, 1413-1422.

(S15)

Where τ is the lag-time, N is the number of molecules in the observation volume, and τ_D is the diffusion time. F represents the amplitude of the relaxation time (τ_R) representing the fraction of molecules in the non-fluorescent state. ω denotes the structure parameter of the observation volume and is given by $\omega = \omega_z/\omega_{xy}$, in which ω_z and ω_{xy} are the longitudinal and transverse radii of the observation volume, respectively. The known diffusion coefficient of Rhodamine 6G in water (426 $\mu m^2 s^{-1}$) was used for calibrating the structure parameter (ω_{xy}~319 nm, ω~5). The confocal volume of our microscope setup is 0.9 fL.

The fluctuation in the fluorescence intensity (and hence FCS data) arises from quenching of the fluorescence of the probe (FAM) by DNA nucleotides (e.g. guanine). In the open conformation of the DNA, the fluorescence probe (FAM) and the quenching groups (e.g. guanine) are far apart and hence the open form is fluorescent. The closed form is non-fluorescent because of rapid electron transfer quenching. If k_+ and k_- denote the rate constants of the formation of non-fluorescent state (open-to-closed inter-conversion) and fluorescent state (closed-to-open inter-conversion), respectively, in the case of two-states at equilibrium (open ↔ closed) one can write,

$$\tau_R = \frac{1}{k_+ + k_-} \text{...........}(S16)$$

$$K_{folding} = \frac{F}{(1-F)} = \frac{k_+}{k_-} \text{.................}(S17)$$

Where $K_{folding}$ is the equilibrium constant and F is the average fraction of molecules in the non-fluorescent state.

$$\text{Evidently,} \quad k_+ = \frac{F}{\tau_R} \text{..................}(S18)$$

Further, we attempted to elucidate the conformation dynamics of the *c-MYC*-(A) and *h-TELO*-(A) in the presence and absence of **BTC**, involving only two states having same diffusion coefficients ($D=D_A= D_B$).Here, the donor molecule of the open state or state A is fluorescent and have a definite quantum yield (i.e. $Q_A= Q$). However, due to FRET, the quantum yield of donor of state B is theoretically zero (i.e. $Q_B = 0$).

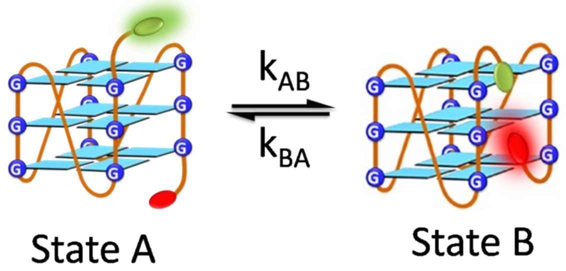

Therefore, the appropriate equation for our case will be[13]

$$G(t)=\frac{1}{\overline{N}}\left(1+\frac{t}{\tau_D}\right)^{-1}\left(1+\frac{t}{\omega^2\tau_D}\right)^{-1/2}\left(1+K\exp\left(-\frac{t}{\tau}\right)\right) \qquad \text{(S19)}$$

The diffusion parameters calculated using this equation is similar to that obtained from equation 1. Actually if we simplify the three dimensional equation S19 for two dimensional case we will get equation 1.

Table S5. Table representing diffusion coefficient (D_t), diffusion time (τ_D), rate constants of donor acceptor contact formation (k_+), dissociation (k_-) of *c-MYC*-(A) and *h-TELO*-(A), the observed amplitude of the donor acceptor contact formation kinetics due to changes of the donor-acceptor distance ($\mathbf{K^C_{obs}}$), and the observed time of donor acceptor contact formation $\boldsymbol{\tau^C_{obs}}$ using equation S19.

System	τ_D (µs)[a]	D_t (µm^2/s)[a]	K^C_{obs}[a]	τ^C_{obs} (µs)[a]	k_+(ms^{-1})	k_- (ms^{-1})
c-MYC-(A)	115	221	1.08	47	11.1 ± 1.1	10.2± 1
c-MYC-(A) + K^+	86	296	0.48	8	40.5 ± 4	84.5±8
c-MYC-(A) + K^+ + **BTC**	80	318	0.94	5	97 ± 10	103 ± 10
c-MYC-(A) + **BTC** (1 eq)	80	318	1.15	6	89.2 ± 9	77.5 ± 8
h-TELO-(A)	106	240	0.46	29	10.8 ± 1.1	23.7 ± 2.5
h-TELO-(A) + K^+	91	280	0.58	9	40.8 ± 4.1	70.3 ± 7
h-TELO-(A) + K^+ + **BTC**	81	314	0.98	7	70.7 ± 7.1	72.2 ± 7
h-TELO-(A) + **BTC** (1 eq)	80	318	0.87	7	66.5 ± 7	76.4 ± 8

[a]±10 %, calculated from the standard deviation of the fitting of each data set.

[13]O. Krichevsky, G. Bonnet Fluorescence Correlation Spectroscopy: the Technique and its Applications. *Rep. Prog. Phys.* 2002, **65**, 251–297.

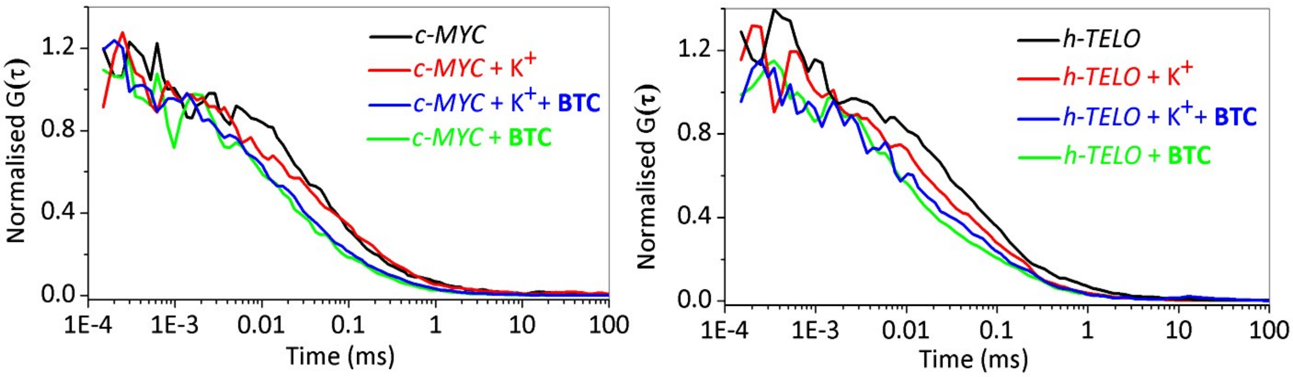

Figure S9. The autocorrelation traces of FAM single labeled *c-MYC*-(B) (left), *h-TELO*-(B) (right) in the presence and absence of K^+ and **BTC**.

Table S6. Table representing diffusion time (τ_D), relaxation time (τ_R) and the fraction of molecules in the non-fluorescent state (F) for FAM labeled *c-MYC*-(B) and *h-TELO*-(B), rate constants of donor acceptor contact formation (k_+) and dissociation ($k\_$) of *c-MYC*-(B) and *h-TELO*-(B) using equation S15**.**

**System**	**τ_D (μs)^b**	**τ_R (μs)^b**	**F**	**k_+ (ms^{-1})^b**	**k_ (ms^{-1})^b**
c-MYC-(B)	115	45	0.5	11.1	11.1
c-MYC-(B) + K^+	86	10	0.34	34	66
c-MYC-(B) + K^+ + **BTC**	80	5	0.51	102	98
c-MYC-(B) + **BTC** (1 equiv.)	80	6	0.50	83.3	83.4
h-TELO-(B)	105	25	0.32	12.8	27.2
h-TELO-(B) + K^+	90	10	0.42	42	58
h-TELO-(B) + K^+ + **BTC**	80	8	0.57	71.25	53.8
h-TELO-(B) + **BTC** (1 equiv.)	85	6	0.47	78.3	88.4
^{b} ±10% SD.					

9.0 Lifetime data

Time correlated single photon counting (TCSPC) measurement: The fluorescence lifetimes of single labeled (donor only) and dual labeled (donor and acceptor) *c-MYC* and *h-TELO* in presence and absence of **BTC** and K^{+} were measured using time-resolved confocal microscopy. In order to measure the fluorescence lifetime, 50 µL (100 nM) of solution was placed on a cover slide. The samples were excited through a water immersion objective (magnification 60X and numerical aperture (NA) ≈ 1.2) with a 470-nm pulsed laser (PicoQuant, IRF ~ 100 ps). An excitation power of ~ 27 µW was used. The emission was collected with the same objective and detected by Micro Photon Devices (MPD-PDM 50CT) through a dichroic beam splitter (HQ490DCXR, Chroma), bandpass filter (e.g. XBPA 510, Asahi Spectra), and 30-µm pinhole for spatial filtering to reject out-of-focus signals. The signal was subsequently processed by the PicoHarp-300 time-correlated, single photon counting module (PicoQuant) to generate TCSPC histogram. The parallel ($I_{\parallel}$, parallel to the polarization of the exciting light) and perpendicular ($I_{\perp}$) components of the fluorescence were separated using a polarizer cube and recorded by using the two detectors (MPDs). $I_{\parallel}$ and $I_{\perp}$ were combined to generate the fluorescence lifetime decays at magic angle conditions as follows,

$$\begin{aligned} I_{magic}(t) &= I_{C}(t)\cos^{2}(54.75°) + I_{\perp}(t)\cdot G\cdot\sin^{2}(54.75°) \\ &= (1/3)\, I_{C}(t) + (2/3)\cdot G\cdot I_{\perp}(t) \end{aligned}$$

G is the correction factor for the difference in the detection efficiency of the two detectors. G factor for this microscope setup was measured by tail fitting of fluorescein[14].

Fluorescence Lifetime measurement: For recording IRF, we used a bare slide and collected the scattered laser light. The FWHM of the IRF for excitation at 470 nm is ~100 ps. The fluorescence decay is deconvoluted using the IRF and DAS6 v6.3 software. Intensity decays can be fitted to the single or multi-exponential model as:

$$I(t) = B + \sum_{i} A_{i} e^{\left(-t/\tau_{i}\right)} \qquad \text{...............} \quad (S20)$$

Where, A represents the fractional amount of fluorophore in each environment and I(t) is the fluorescence intensity at time t. B is the pre-exponential factor.

[14] S. T. Hess, E. D. Sheets, A. Wagenknecht-Wiesner, A. A. Heikal, *Biophys. J.* 2003, **85**, 2566-2580.

The R_{DA} between donor and acceptor labels attached to *c-MYC*-(A) and *h-TELO*-(A) were estimated from the average FRET efficiency (ε_{avg}) as,

$$\varepsilon_{avg} = 1 - \frac{\tau_{DA}}{\tau_D} = [1 + (\frac{R_{DA}}{R_0})^6]^{-1} ....(S21)$$

Where, R_0 represents the Förster distance, the average lifetime (τ_{avg}) of donor labeled *c-MYC*-(B) and *h-TELO*-(B) is represented as "τ_D" and donor-acceptor labeled *c-MYC*-(A) and *h-TELO*-(A) is τ_{DA}.

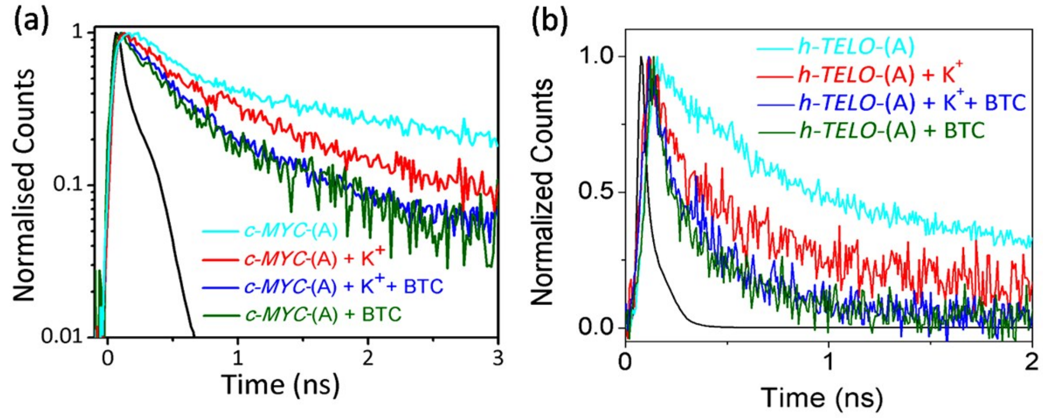

Figure S10. Fluorescence decay profiles of the (a) donor attached to dual labeled *c-MYC*-(A) alone, in the presence of K^+, in the presence of **BTC**, in the presence of K^+ and **BTC** and (b) donor attached to dual labeled *h-TELO*-(A) alone, in the presence of K^+, in the presence of **BTC**, and in the presence of K^+ and **BTC** was measured using a time-resolved fluorescence microscope fitted with confocal optics. The black line corresponds to the instrument response function.

We determined the fitting model based on best fit. For instance, we have fitted *c-MYC*-(A) to tri-exponential decay because we obtained best fit (residuals) in tri-exponential decay model as shown below,

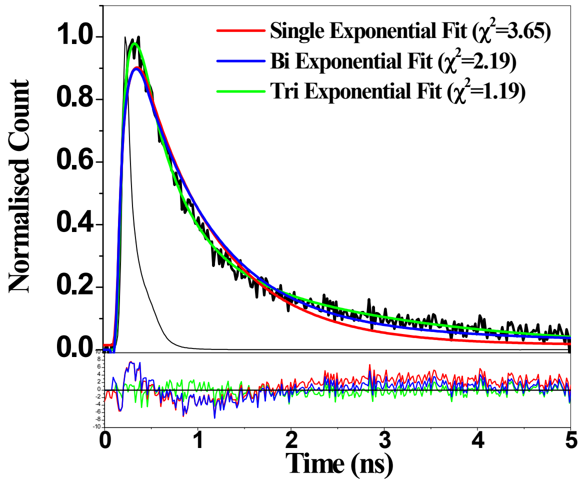

Figure S11. Representative fit of fluorescence decay profile of *c-MYC*-(A) to tri-exponential decay. Lower panel shows the residual of the fitting result.

Table S7. Lifetime parameters of *c-MYC* at λ_{em}= 510 nm.

System		τ_1 (ns) [a]	A_1	τ_2 (ns) [a]	A_2	τ_3 (ns) [a]	A_3	τ_{avg} (ns) [a]	ε_{avg} [a]	[R_{DA}] [a] (Å)
c-MYC-(B)	τ_D	--		--		2.75		2.75	0.68	48.5
c-MYC-(A)	τ_{DA}	0.13	0.02	0.4	0.72	2.25	0.26	0.88		
c-MYC-(B)+ K^+	τ_D			1.0	0.25	3.6	0.75	2.95	0.79	44.1
c-MYC-(A)+ K^+	τ_{DA}	0.13	0.46	0.53	0.45	3.45	0.09	0.61		
c-MYC-(B)+K^+ + **BTC**	τ_D			1.5	0.35	4.85	0.65	3.68	0.84	41.7
c-MYC-(A)+K^+ +**BTC**	τ_{DA}	0.13	0.68	0.8	0.24	3.7	0.08	0.57		
c-MYC-(B) + **BTC**	τ_D					3.7		3.7	0.88	39.4
c-MYC-(A) + **BTC**	τ_{DA}	0.13	0.67	0.72	0.29	3.67	0.04	0.44		

A_1, A_2, A_3 are the pre-exponential factors which represent the fraction of fluorescent species having lifetimes τ_1, τ_2, τ_3. τ_{avg} is the average lifetime. ε_{avg} represents the average FRET efficiency.[a]± 10 % SD.

Table S8. Lifetime parameters of *h-TELO* at λ_{em}= 510 nm.

System		τ_1 (ns) [a]	A_1	τ_2 (ns) [a]	A_2	τ_{avg} (ns) [a]	ε_{avg} [a]	R_{DA} (Å) [a]
h-TELO	B	--		2.05		2.05	0.49	55.37
	A	0.2	0.26	1.27	0.74	0.99		
h-TELO +K^+	B	--		2.95		2.95	0.80	43.65
	A	0.18	0.6	1.22	0.4	0.596		
h-TELO + K^++**BTC**	B	--		2.12		2.12	0.83	42.23
	A	0.05	0.72	1.2	0.28	0.37		
h-TELO + **BTC**	B	--		1.4		1.4	0.85	41.19
	A	0.07	0.64	0.45	0.36	0.21		

[a]± 10 % SD.

10.0 CD spectroscopy: CD spectra were recorded on a JASCO J-815 spectrophotometer by using a 1 mm path length quartz cuvette. Aliquots of **BTC** were added stepwise to pre-annealed *c-MYC*-(D) 5′-($TGAG_3TG_3TAG_3TG_3TA_2$)-3′ and *h-TELO*-(D) 5′-($G_3TTAG_3TTAG_3TTAG_3$)-3′ quadruplex sequences in Tris•HCl (10 mM) buffer at pH 7.4 with or without KCl (100 mM). The CD spectra were recorded upon incremental addition of **BTC** to the *c-MYC*-(D) and *h-TELO*-(D) quadruplex sequences. The

DNA concentrations used were 10μM. For the CD analysis of FRET probes, 5 μM of *c-MYC*-(A) and *h-TELO*-(A) were annealed in Tris•HCl (10 mM) buffer with KCl (100 mM) at pH 7.4. The CD spectra represent an average of three scans and were smoothed and zero corrected. Final analysis and manipulation of the data was carried out by using Origin 8.0.

The CD spectrum of *c-MYC*-(D) sequence exhibited the characteristic positive peak at 270 nm and a negative peak at 240 nm for parallel G-quadruplex structure in K^+ buffer (**Figure S12 a-b**).[15] The CD spectrum of *h-TELO*-(D) sequence showed the characteristic positive peak at 290 nm and a shoulder at 260 nm for mixed parallel-antiparallel type G-quadruplex conformation in K^+ buffer (**Figure S12 c**).[15] In the absence of K^+, the CD spectrum of *h-TELO*-(D) showed a positive peak at ~ 298 nm, a negative peak at ~ 265 nm and a small positive peak at ~244 nm, which is characteristic for the antiparallel G-quadruplex conformation **(Figure S12 d)**. The CD spectra of dual labeled *c-MYC*-(A) and *h-TELO*-(A) suggested that the attachment of the fluorophores does not change the conformation of G-quadruplexes (**Figure S13 a-b**).

Addition of **BTC** to the *c-MYC*-(D) in the presence or absence of K^+ ions hardly altered the characteristic CD peaks, indicating that **BTC** did not induce any significant conformational change to the *c-MYC*-(D) parallel G-quadruplex structure. The CD peaks of *h-TELO*-(D) in K^+ were marginally affected after the addition of **BTC**. Upon addition of **BTC** to the *h-TELO*-(D) in the absence of K^+, the peak at 298 nm (antiparallel conformation) was shifted to ~ 290 nm. These results indicate that the ligand bound *c-MYC*-(D) and *h-TELO*-(D) quadruplex conformations are similar in the presence and absence of K^+.

[15] J. Dash, R. Nath Das, N. Hegde, G. D. Pantoş, P. S. Shirude, S. Balasubramanian, *Chem Eur. J.* 2012, **18**, 554-564.

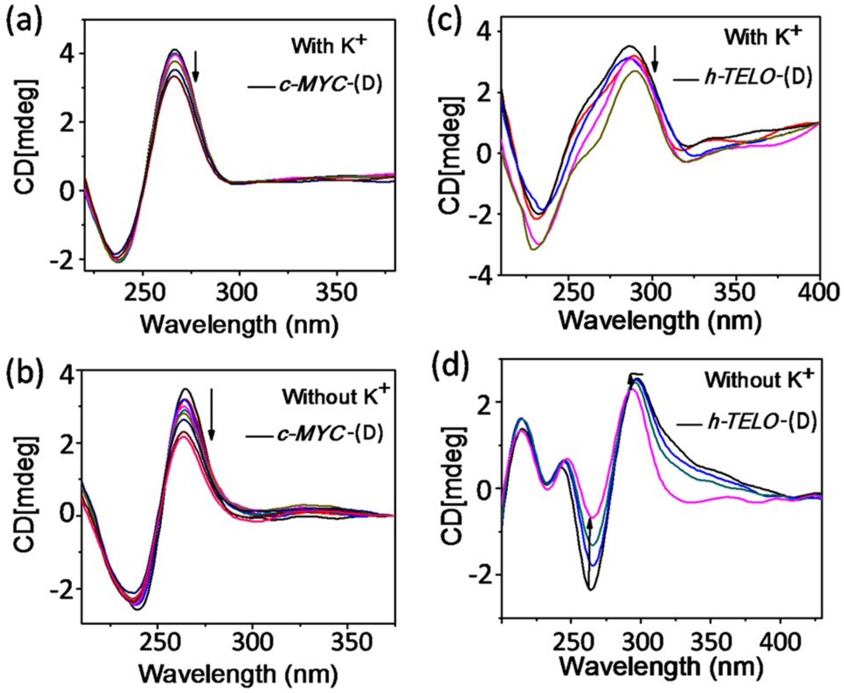

Figure S12. CD spectra of a solution of (a) *c-MYC*-(D) (10 μM) in 10 mM Tris-HCl buffer (pH 7.4) with KCl (100 mM), titrated with **BTC** (0–4 equiv);(b) *c-MYC*-(D) (10μM) in 10 mM Tris-HCl buffer (pH 7.4) without KCl (100 mM), titrated with **BTC** (0–4 equiv);(c) *h-TELO*-(D) (10 μM) in 10 mM Tris-HCl buffer (pH 7.4) with KCl (100 mM), titrated with **BTC** (0–4 equiv); (d) *h-TELO*-(D) (10 μM) in 10 mM Tris-HCl buffer (pH 7.4) without KCl (100 mM), titrated with **BTC** (0–4 equiv).

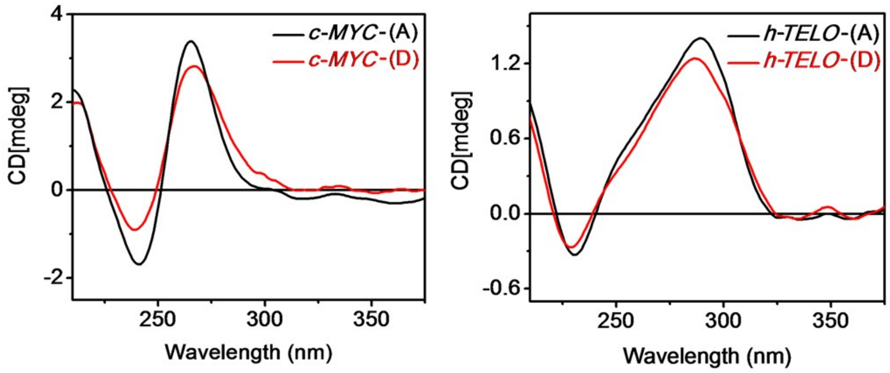

Figure S13. CD spectra of a solution of (a) dual-labeled and unlabeled *c-MYC* in 10 mM Tris-HCl buffer (pH 7.4) with KCl (100 mM); (b) dual-labeled and unlabeled *h-TELO* in 10 mM Tris-HCl buffer (pH 7.4) with KCl (100 mM).

11.0 NMR spectroscopy: The G-rich nuclease hypersensitivity element NHE III_1 located upstream of the *c-MYC* promoter contains five consecutive G-tracts and is able to fold into parallel-stranded G-quadruplex structures. This element is highly dynamic and can form different loop isomers involving

different G-tracts.[16,17,18,19,20,21] The *c-MYC*–(D) sequence used for NMR studies derives from the four G-tracts at the 3′-end of NHE III_1 and its solution structure has been determined by Ambrus *et al.*[20] The G-rich sequence in human telomere forms a propeller-type parallel-stranded G-quadruplex structure in K^+ containing crystal[22]. However, the quadruplex structures with (3 + 1) mixed parallel and antiparallel strands connected by propeller and lateral edgewise loops are the predominant species present in the in K^+ containing solution[23]. The NMR structure of the *h-TELO* DNA sequence used in this study [*h-TELO*-(D)] has not been solved until now. However, it has been shown that truncated telomeric sequences without 5′-flanking nucleotides, like *h-TELO,* can form a basket-type quadruplex with only two G-tetrads connected by lateral edgewise and diagonal loops.[24,25] By comparison of the imino pattern of *h-TELO* with the literature data,[20,21] we tentatively assigned the imino proton signals of *h-TELO* as reported in **Figure 4**.

The *c-MYC*-(D) 5′-(TGAG_3TG_3TAG_3TG_3TAA)-3′ and *h-TELO*-(D) 5′-(G_3TTAG_3TTAG_3TTAG_3)-3′ DNA used for NMR experiments were purchased by Eurofins MWG Operon (Ebersberg, Germany) as HPSF® (High Purity Salt Free) purified oligos and further purified *via* HPLC.

---

[16] J. Seenisamy, E. M. Rezler, T. J. Powell, D. Tye, V. Gokhale, C. S. Joshi, A. Siddiqui-Jain, L. H. Hurley, *J. Am. Chem. Soc*. 2004, **126**, 8702-8709.

[17] E. Hatzakis, K. Okamoto, D. Yang, *Biochemistry* 2010, **49**, 9152-9160.

[18] A. T. Phan, Y. S. Modi, D. J. Patel, *J. Am. Chem. Soc*. 2004, **126**, 8710-8716.

[19] A. T. Phan, V. Kuryavyi, H. Y. Gaw, D. J. Patel, *Nat Chem Biol.* 2005, **1**, 167-173.

[20] A. Ambrus, D. Chen, J. Dai, R. A. Jones, D. Yang, *Biochemistry* 2005, **44**, 2048-2058.

[21] R. I. Mathad, E. Hatzakis, J. Dai, D. Yang, *Nucleic Acids Res.* 2011, **39**, 9023-9033.

[22] G. N. Parkinson, M. P. H. Lee, S. Neidle, *Nature* 2002, **417**, 876-880.

[23]A. T. Phan, K. N. Luu, D. J. Patel, *Nucleic Acids Res.* 2006, **34**, 5715-5719.

[24] K. W. Lim, S. Amrane, S. Bouaziz, W. Xu, Y. Mu, D. J. Patel, K. N. Luu, A. T. Phan, *J. Am. Chem. Soc*. 2009, **131**, 4301-4309.

[25] Z. Zhang, J. Dai, E. Veliath, R. A. Jones, D. Yang, *Nucleic Acids Res.* 2010, **38**, 1009-1021.

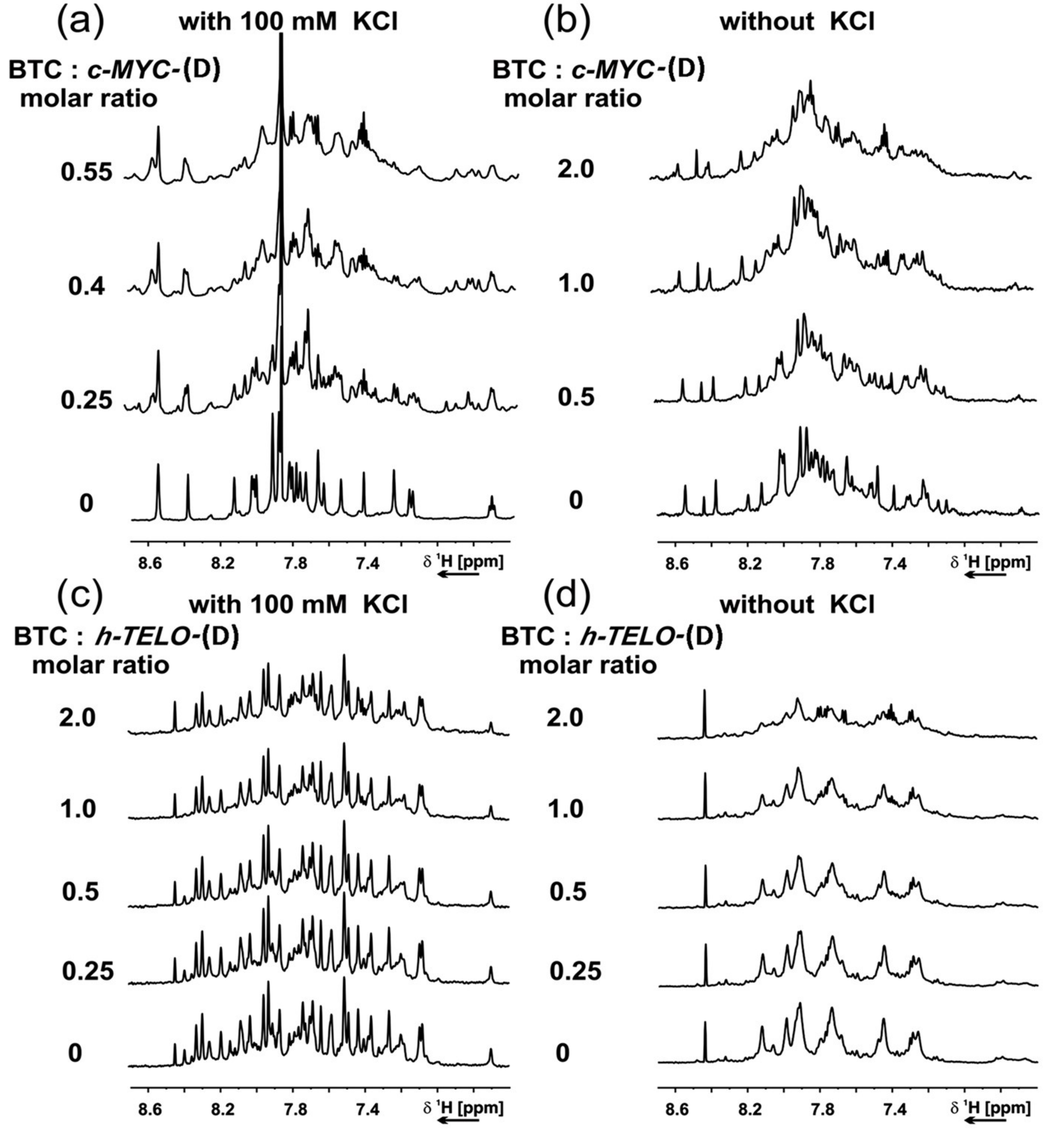

Figure S14. NMR titration of *c-MYC*-(D)and *h-TELO*-(D) with ligand **BTC**, aromatic region of the 1D ^{1}H NMR spectrum of *c-MYC*-(D) in the presence of increasing amount of **BTC**, with (a) or without (b) additional 100 mM KCl. Aromatic region of the 1D ^{1}H NMR spectrum of *h-TELO*-(D) in the presence of increasing amount of **BTC**, with (c) or without (d) additional 100 mM KCl. Experimental conditions: 100 μM DNA, 25 mM Tris·HCl buffer (pH 7.4), 298 K, 600 MHz.

NMR samples were referenced with 2,2-dimethyl-2-silapentane-5-sulphonate (DSS) and prepared in 25 mM Tris·HCl (pH 7.4) containing 100 mM KCl, if not otherwise specified. Assignment of *c-MYC*-(D) was performed according to the one reported by Ambrus *et al.*[20] while assignment of *h*-

TELO-(D) was attempted on the basis of the one reported for analogous sequences by Lim *et al.*[24] and Zhang *et al.*[25] ^{1}H NMR spectra were recorded with gradient-assisted excitation sculpting[26] or jump-return-Echo[27] for water suppression at 600 MHz and 298 K.

In the absence of K^+, the aromatic proton signals of *h-TELO*-(D) (**Figure S14d**) are not as dispersed as observed in the presence of K^+ (**Figure S14c**) and resemble mainly the signal pattern of an unfolded oligonucleotide.

---

[26] T. L. Hwang, A. J. Shaka, *J. Magn. Reson., Ser B,* **1995,** *112*, 275–279.

[27] V. Sklenář, A. Bax, *J. Magn.Reson.***1987**, *74*, 469–479.
